# The bacterial biome of ticks and their wildlife hosts at the urban–wildland interface

**DOI:** 10.1099/mgen.0.000730

**Published:** 2021-12-16

**Authors:** Siobhon L. Egan, Casey L. Taylor, Peter B. Banks, Amy S. Northover, Liisa A. Ahlstrom, Una M. Ryan, Peter J. Irwin, Charlotte L. Oskam

**Affiliations:** ^1^​ Harry Butler Institute, Murdoch University, Murdoch, Western Australia, 6150, Australia; ^2^​ School of Life and Environmental Sciences, The University of Sydney, Camperdown, New South Wales, 2006, Australia; ^3^​ School of Veterinary Medicine, Murdoch University, Murdoch, Western Australia, 6150, Australia; ^4^​ Elanco Animal Health, Macquarie Park, New South Wales, 2113, Australia

**Keywords:** tick, microbiome, wildlife, sylvatic cycle, tick-borne diseases

## Abstract

Advances in sequencing technologies have revealed the complex and diverse microbial communities present in ticks (Ixodida). As obligate blood-feeding arthropods, ticks are responsible for a number of infectious diseases that can affect humans, livestock, domestic animals and wildlife. While cases of human tick-borne diseases continue to increase in the northern hemisphere, there has been relatively little recognition of zoonotic tick-borne pathogens in Australia. Over the past 5 years, studies using high-throughput sequencing technologies have shown that Australian ticks harbour unique and diverse bacterial communities. In the present study, free-ranging wildlife (*n*=203), representing ten mammal species, were sampled from urban and peri-urban areas in New South Wales (NSW), Queensland (QLD) and Western Australia (WA). Bacterial metabarcoding targeting the 16S rRNA locus was used to characterize the microbiomes of three sample types collected from wildlife: blood, ticks and tissue samples. Further sequence information was obtained for selected taxa of interest. Six tick species were identified from wildlife: *Amblyomma triguttatum*, *Ixodes antechini*, *Ixodes australiensis*, *Ixodes holocyclus*, *Ixodes tasmani* and *Ixodes trichosuri*. Bacterial 16S rRNA metabarcoding was performed on 536 samples and 65 controls, generating over 100 million sequences. Alpha diversity was significantly different between the three sample types, with tissue samples displaying the highest alpha diversity (*P*<0.001). *Proteobacteria* was the most abundant taxon identified across all sample types (37.3 %). Beta diversity analysis and ordination revealed little overlap between the three sample types (*P*<0.001). Taxa of interest included *Anaplasmataceae *, *Bartonella *, *Borrelia *, *Coxiellaceae *, *Francisella *, *Midichloria *, *Mycoplasma* and *Rickettsia *. *Anaplasmataceae* bacteria were detected in 17.7% (95/536) of samples and included *Anaplasma *, *Ehrlichia* and *Neoehrlichia* species. In samples from NSW, ‘*Ca*. Neoehrlichia australis’, ‘*Ca*. Neoehrlichia arcana’, *Neoehrlichia* sp. and *Ehrlichia* sp. were identified. A putative novel *Ehrlichia* sp. was identified from WA and *Anaplasma platys* was identified from QLD. Nine rodent tissue samples were positive for a novel *Borrelia* sp. that formed a phylogenetically distinct clade separate from the Lyme *Borrelia* and relapsing fever groups. This novel clade included recently identified rodent-associated *Borrelia* genotypes, which were described from Spain and North America. *Bartonella* was identified in 12.9% (69/536) of samples. Over half of these positive samples were obtained from black rats (*Rattus rattus*), and the dominant bacterial species identified were *Bartonella coopersplainsensis* and *Bartonella queenslandensis *. The results from the present study show the value of using unbiased high-throughput sequencing applied to samples collected from wildlife. In addition to understanding the sylvatic cycle of known vector-associated pathogens, surveillance work is important to ensure preparedness for potential zoonotic spillover events.

## Data Summary

Sequence data have been deposited in relevant data repositories (see above). Additional information is available within supplementary files (available in the online version of this article) including links to electronic files which have been deposited on FigShare: https://doi.org/10.6084/m9.figshare.14363627.v1. Code used for analysis and RData files used for bioinformatics and statistical analysis are available at https://github.com/siobhon-egan/wildlife-bacteria and https://githubcom/siobhon-egan/wildlife-ticks.

The authors confirm all supporting data, code and protocols have been provided within the article or through supplementary data files.

**Impact Statement**

Globally, the incidence and distribution of tick-borne diseases is increasing. In contrast, there is currently limited data available regarding tick-borne pathogens in Australia. This research employed bacterial 16S metabarcoding on wildlife blood and tissue samples and their ticks. It aimed to understand the sylvatic cycle of microbes and identify potential pathogens. This study identified several recently described and novel microbes from both wildlife and ticks. It identified *Neoehrlichia* and *Ehrlichia* species from native wildlife and the introduced black rat. It describes the first identification of the rodent-associated *Borrelia* clade in Australia; this unique clade is distinct from both the Lyme *Borrelia* and relapsing fever groups. Black rats were identified as an important reservoir of both endemic and novel microbes. Importantly, these findings provide support for the absence of northern hemisphere tick-borne pathogens. In conclusion, it is likely that any potential human tick-borne pathogen(s) in Australia are probably endemic and distinct from currently described pathogens from the northern hemisphere.

## Introduction

Ticks carry a diverse range of infectious microbes such as viruses, piroplasms, spirochaetes and *Rickettsiales* [1]. Globally recognized infectious human tick-borne diseases include Lyme borreliosis, ehrlichiosis, babesiosis, tick-borne encephalitis and Powassan viral disease. Additionally, a tick bite can cause reactions such as paralysis and anaphylaxis [2]. The incidence of tick-borne diseases is rapidly increasing in both prevalence and geographical distribution [3]. The sylvatic cycles of tick-borne pathogens in the northern hemisphere are generally well understood with respect to competent tick vectors and the reservoir hosts (e.g. *Babesia microti* [4] and *Borrelia burgdorferi sensu lato* [5]). The value of wildlife health surveillance as a tool for the detection of emerging zoonotic infectious disease has been demonstrated in outbreaks historically, such as malaria [6], and more recently with SARS-CoV-2 [7].

In Australia, three human tick-borne diseases are recognized: Queensland tick typhus (* Rickettsia australis *), spotted fever (* Rickettsia honei* subspp.) and Q fever (* Coxiella burnetii *) [8]. Over the past decade there have been increasing concerns about unidentified tick-borne illnesses (syn. Lyme-like disease) in Australia [9], recently designated ‘Debilitating Symptom Complexes Attributed To Ticks’ (DSCATT) by the Australian Government Department of Health [10]. Presently, 74 tick species have been described in Australia [11, 12], including five introduced species, of which 22 species have been identified biting humans [13]. However, data show that *Ixodes holocyclus* and *Amblyomma triguttatum* account for >90 % of human tick bites, with the remainder of reports largely attributed to *Ixodes tasmani*, *Haemaphysalis bancrofti*, *Haemaphysalis longicornis* and *Bothriocroton hydrosauri* [13]. Small to medium-sized mammals are important in maintaining these tick populations, in particular during the immature life stages [14–16]. While data on the incidence of human tick bites in Australia are limited, evidence suggests that tick bites are common in peri-urban areas, including residential yards (C. Taylor, pers. comm.).

Over the past decade, due to new technologies there is mounting evidence that Australian ticks harbour a unique array of microbes [17, 18]. In addition to these molecular studies, more targeted screening of potential reservoirs such as dogs [19] and sea bird ticks [20] have added to the growing body of evidence suggesting that human tick-borne pathogens described in the northern hemisphere are not endemic in Australia. Some of the microbes that have recently been identified from Australian ticks, however, include novel species of *Borrelia* [21, 22], *Ehrlichia* [23], *Neoehrlichia* [24], *Babesia* and *Theileria* [25, 26] and several viruses [27, 28]. Little is known about the sylvatic life cycle of these microbes or their wildlife species reservoir hosts. Even for the few tick-borne pathogens recognized in Australia (e.g. *Rickettsia* spp.), much of this information remains elusive.

For a tick-borne disease to become established it requires a temporal and spatial overlap between the microbe (pathogens), competent vector tick(s), and one or more vertebrate reservoir hosts. In the northern hemisphere studies have shown that there are important differences in the role of host species in maintaining the sylvatic cycle of tick-borne pathogens [29–31]. Broadly, reservoir hosts can be classified as amplification and dilution hosts. Amplification hosts, in most cases, are small mammals that host immature ticks (larvae or nymph stages) and increase the environmental density of infected nymphs [29]. In contrast, dilution hosts are inefficient reservoirs of the microbial agent(s) [32, 33]. An additional factor is the role of hosts throughout the tick life cycle. Larger animals are considered important in maintaining adult stages of the tick: white-tailed deer (*Odocoileus virginianus*) and *Ixodes scapularis* [34] for example. In the Australian context it is generally assumed that small mammals such as bandicoots, possums and rodents are likely to be reservoirs for tick-associated microbes. In turn, larger mammals such as kangaroos and wallabies may then play an important role in maintaining tick populations, filling a similar ecological niche as deer in the northern hemisphere.

While studies have used molecular tools to investigate microbes present within ticks in Australia [20, 24, 25], very little research has been conducted at the vertebrate host level to further elucidate the sylvatic cycle of tick-transmitted bacteria. Bacterial amplicon sequencing is valuable in the setting of wildlife epidemiology surveys where investigations are not confined to existing presumptions of microbes [35], but such studies of the ticks alone, without concurrent analysis of vertebrate host tissues, cannot distinguish infectious components of the tick’s total microbiome from those that are non-transmissible. Therefore, the search for tick-associated microbes in vertebrate host(s) is the next logical piece of information required to untangle the complex dynamics of tick-borne pathogens. In addition to novel pathogen discovery, high-throughput sequencing technologies applied concomitantly to tick and vertebrate host tissues can provide insights into the dynamics of microbial epidemiology and interactions within landscapes [36]. Identification of microbes may inform our understanding of the tick-borne disease risk and potential differences among temporal and spatial gradients.

The present study used metabarcoding to explore the bacterial communities present in Australian wildlife and their ticks, with a focus on bacteria related to known (vector-borne) pathogens, to provide insights into the sylvatic life cycle of tick-associated organisms. This research was conducted in an ecological zone referred to as the urban–wildland interface, where there is a transition between wilderness and land developed by human activity – an area where a built environment meets or intermingles with a natural environment.

## Methods

### Study sites and sample collection

#### Small mammal trapping

**Fig. 1.**
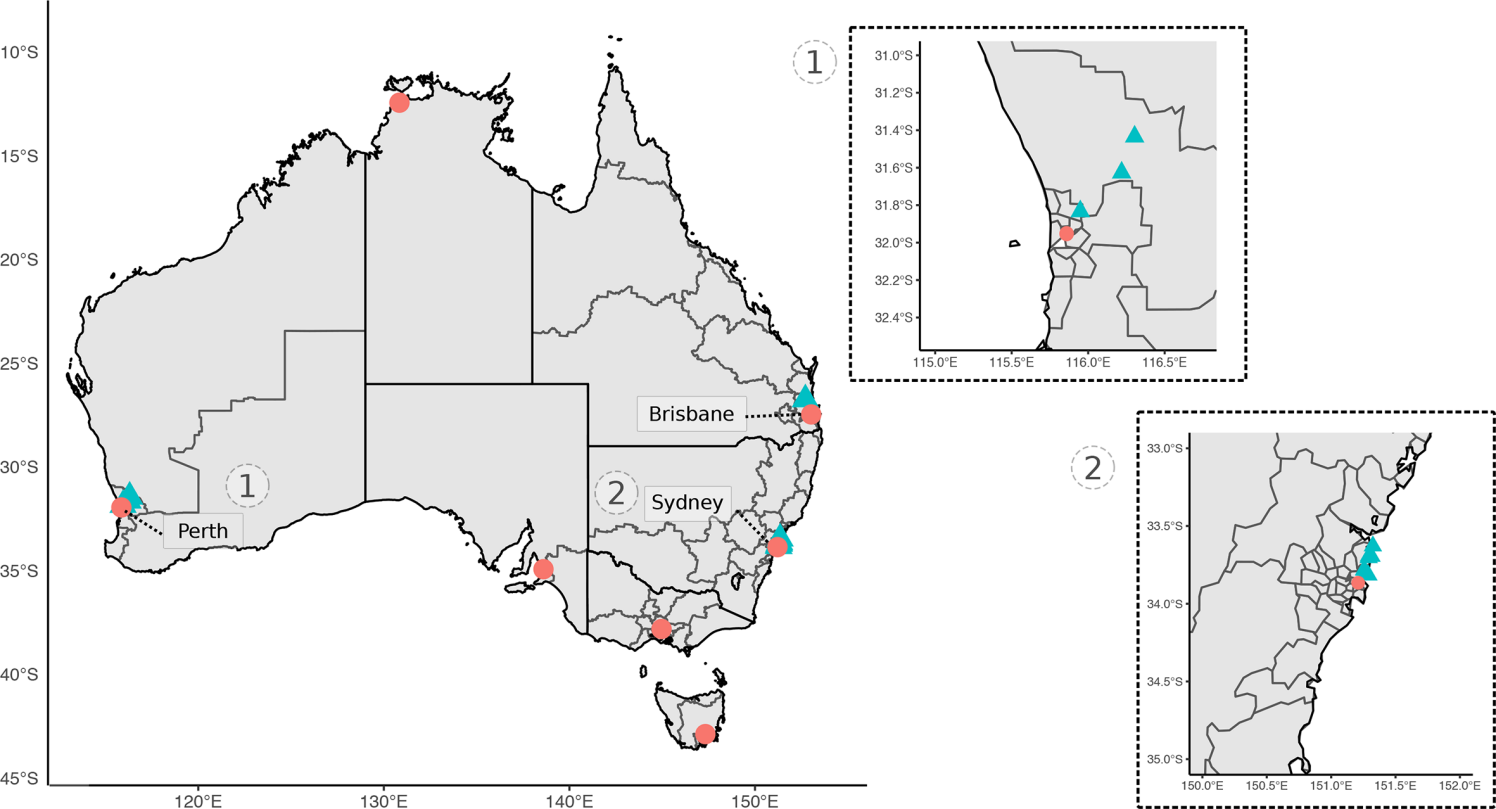
Map of study sites for collection of wildlife samples used in bacterial profiling. Sampling sites are denoted by blue triangles and capital cities by pink circles (for geographical reference). Insert maps of sites in (1) Perth, Western Australia, and (2) Sydney, New South Wales.

Small mammal trapping was performed at various sites in Perth, Western Australia, and Sydney, New South Wales (Fig. 1). Each site was targeted in urban and peri-urban areas to reflect the urban–wildland interface. Elliot and cage traps were set and baited with universal bait (rolled oats, peanut butter and sardines). Traps were set at dusk and cleared at sunrise over three or four consecutive nights. To ensure a comprehensive assessment and sampling, target mammals were briefly anaesthetized in the field using isoflurane (I.S.O., 1 ml ml^−1^) vaporized in medical oxygen. Animals were weighed and examined systematically for ectoparasites. Up to 1 ml of blood was collected from either the caudal (tail) vein, femoral vein or ear capillary and stored in Mini Collect EDTA tubes. If practical, a 2 mm punch biopsy was taken at the tick bite site; where possible, a biopsy was taken from the ear and stored in RNAlater or 80% ethanol. Animals were systematically examined for ectoparasites, which were removed and stored in 80 % ethanol. Animals were recovered from anaesthesia by providing medical oxygen, and once fully alert were released at the trap point. An individual mark was applied to identify animals by using either a microchip administered subcutaneously or a unique patch of hair was removed. The number of animals and samples analysed, and locations from the present study are available in Table 1 and Fig. 1 respectively.

**Table 1.** Animal sampling numbers for bacterial microbiome sequencing: number of individual animals (*n*) by host species with number of genomic DNA samples analysed in parentheses

Common name	Scientific name	*n*	Blood	Tick	Tissue
Black rat	*Rattus rattus*	*88*	68 (84)	54 (79)	71 (80)
Brown antechinus	*Antechinus stuartii*	*5*	0 (0)	4 (6)	2 (2)
Brush-tailed possum	*Trichosurus vulpecula*	*27*	18 (21)	15 (33)	16 (20)
Bush rat	*Rattus fuscipes*	*3*	2 (2)	3 (4)	3 (3)
Chuditch	*Dasyurus geoffroii*	*22*	22 (24)	1 (1)	9 (9)
Deer	*Rusa timorensis*	*3*	0 (0)	0 (0)	3 (6)
Long-nosed bandicoot	*Perameles nasuta*	*44*	20 (25)	31 (60)	40 (44)
Quenda	*Isoodon fusciventer*	*2*	1 (1)	2 (2)	0 (0)
Rabbit	*Oryctolagus cuniculus*	*7*	0 (0)	7 (20)	7 (7)
Swamp rat	*Rattus lutreolus*	*2*	1 (1)	0 (0)	1 (1)
**Total**		***203***	**132** (159)	**117** (205)	**152** (172)

### Opportunistic collection

In a small number of cases, animal carcasses were also obtained opportunistically. These specimens were collected through incidental findings during fieldwork or from situations where animals were humanely euthanized in accordance with animal ethics and state and federal guidelines.

### Tick identification

Ticks were collected and stored in tubes containing 70–90% ethanol. Samples were visualized using an Olympus SZ61 stereomicroscope (Olympus) with an external Schott KL 1500 LED (Schott) light source. Photographs of tick specimens were taken using an Olympus SC30 digital camera (Olympus) and analysis getIT software (Olympus). Instar, sex and species were identified using a combination of available morphological keys and species descriptions [37–41].

### DNA extractions

Total genomic DNA (gDNA) was extracted from 200 μl of blood using a MasterPure DNA purification kit (EpicentreBiotechnologies) following the manufacturer’s recommendations. Where 200 μl of blood was not available, sterile DNA-free PBS was used to make samples up to 200 μl. Genomic DNA was eluted in 30–40 μl of TE buffer and stored at −20 °C until further processing.

Tissue samples (skin and spleen) were first rinsed in sterile, DNA-free PBS and cut into small pieces (<1–2 mm) using a sterile scalpel blade. Samples were homogenized in 180 μl of buffer ATL and 20 μl of proteinase K was added and incubated at 56 °C for ~16 h. gDNA was extracted using the QIAamp DNA Mini Kit (Qiagen) following the manufacturer’s protocols with the exception that the final elution volume was decreased to 40–50 μl to increase gDNA yield.

Once ticks were identified, they were surface-sterilized by washing in 10% hypochlorite solution, rinsed in 70% ethanol and DNA-free PBS, and then air-dried. Genomic DNA was extracted using the DNeasy Blood and Tissue kit (Qiagen) for adults, or the QIAamp DNA Mini Kit (Qiagen) for nymphs and larvae. Due to the large number of immature tick stages collected from some animals and the expected low DNA yield, up to five specimens were pooled for extraction based on host, instar, engorgement status and species identification as determined by morphological methods. Ticks were placed in a 2 ml safe lock Eppendorf tube with a 5 mm steel bead, frozen in liquid nitrogen for 1 min and homogenized by shaking at 40 Hz in a Tissue Lyser LT (Qiagen). Final elution of DNA was adjusted according to tick size, and 30–150 μl of AE buffer was added to the silicon membrane.

Extraction controls (EXBs) consisting of 200 μl sterile DNA-free PBS were included randomly in each extraction batch (total EXBs=34). Blood and tissue samples were extracted in a separate laboratory, away from where ticks were processed, to avoid cross-contamination between sample types. Sterile procedures were followed throughout the laboratory process.

### Metabarcoding sequencing

A high-throughput metabarcoding approach was used to sequence samples using the Illumina MiSeq platform. Libraries were built following the 16S Metagenomic Sequencing Library Preparation (Illumina Part no. 15044223 Rev. B), with amplicon PCR primers containing Illumina MiSeq adapter sequences (Table 2). A total of 536 samples from 203 individuals (159 blood, 205 tick pools and 172 tissue samples) underwent bacterial profiling.

**Table 2.** List of sequences used for metabarcoding and target PCRs to screen blood, tissue and tick samples for bacteria References: 1, Gofton *et al*. [17]; 2, Turner *et al*. [144]; 3, Lopez *et al*. [145]; 4, Caporaso *et al*. [146]; 5, Beard *et al*. [147]; 6, Anderson *et al*. [148]; 7, Paddock *et al*. [149]; 8, Kawahara *et al*. [82]; 9, Beati and Keirans 2001 [42].

Primer	Sequence (5′−3′)	Reference
**Bacteria 16S rRNA**		
27F-Y	AGAGTTTGATCCTGGCTYAG	[1]
338R	TGCTGCCTCCCGTAGGAGT	[2]
338F	ACTCCTACGGGAGGCAGCAG	[3]
806R	GGACTACHVGGGTWTCTAAT	[4]
*** Bartonella* 16S rRNA/ITS**		
438s	GGTTTTCCGGTTTATCCCGGAGGGC	[5]
1100as	GAACCGACGACCCCCTGCTTGCAAAGC	[5]
*** Anaplasmataceae* 16S rRNA**		
EC9	TACCTTGTTACGACTT	[6]
EC12	TGATCCTGGCTCAGAACGAACG	[7]
A171a	GCGGCAAGCCTCCCACAT	[8]
IS58-1345r	CACCAGCTTCGAGTTAAACC	[8]
**Ixodida 12S rRNA**		
T1B	AAACTAGGATTAGATACCCT	[9]
T2A	AATGAGAGCGACGGGCGATGT	[9]

Bacterial 16S rRNA libraries were built targeting the 16S hypervariable region 1–2 using primers 27F-Y and 338R [17]. Reactions were carried out in 25 μl volumes each containing: 1× buffer (KAPA Biosystems), 1.5 mM MgCl_2_, 0.4 mg ml^−1^ BSA (Fisher Biotech), 0.4 μM of each primer, 0.25 mM of each dNTP, 0.5 U of Taq (KAPA Biosystems) and 2 μl of gDNA. Thermal cycling conditions were as follows: 95 °C for 5 min, followed by 35 cycles of 95 °C for 30 s, 55 °C for 30 s, 72 °C for 45 s; and a final extension of 72 °C for 5 min. For a subset of samples where microbes of interest were identified after initial screening at the v1–2 hypervariable region, additional bacterial 16S rRNA libraries targeting the v3–4 hypervariable region were also prepared. Primers 338F and 806R were used to target an ~450 bp product. Reactions and thermal cycling conditions were carried out as per the 27F-Y/338R assay, except for an increased concentration of MgCl_2_ to 2.0 mM.

To confirm morphological identification, tick gDNA underwent an amplicon metabarcoding approach targeting the 12S rDNA gene. Pan-Ixodida primers T1B/T2A [42] were used to amplify an ~370 bp product of the 12S rRNA locus. Reactions were carried out as per the bacteria 27F-Y/338R assay, except only 1 μM of tick gDNA template was added. Thermal cycling conditions were as follows: 95 °C for 5 min, followed by five cycles of 95 °C for 15 s, 51 °C for 30 s, 68 °C for 30 s; 25 cycles of 95 °C for 30 s, 53 °C for 30 s, 72 °C for 1 min; and a final extension of 72 °C for 5 min.

Amplicon PCR products were then indexed using the Nextera XT DNA library preparation kit in 25 μl volumes following the manufacturer’s recommendations. All PCRs included no-template controls (NTC; total=31) during each reaction set up and PCRs were performed under strict laboratory conditions. Amplicons were then dual-indexed using the Nextera XT index kit. Reactions were performed in 25 μl volumes following the manufacturer’s recommendations. Libraries were purified with Axygen PCR clean up beads and quantified using a Qubit High Sensitivity dsDNA assay kit (Thermo Fisher Scientific) and pooled in equimolar amounts. Libraries were shipped to the Australian Genomic Research Facility (Melbourne, Australia) for final QC and sequenced on an Illumina MiSeq using v2 chemistry (2×250 paired-end).

### Bioinformatics

Metabarcoding 16S rRNA sequence data were analysed using Quantitative Insights into Microbial Ecology (QIIME 2 2020.11) [43]. Briefly, raw sequences were demultiplexed and quality filtered using the q2-demux plugin, followed by denoising using DADA2 (via q2-dada2) [44] resulting in amplicon sequence variations (ASVs) (i.e. 100% identical operational taxonomic units or OTUs [45]). Bacterial data taxonomy was assigned to ASVs using the q2‐feature‐classifier [46] classify‐sklearn naive Bayes taxonomy classifier against the silva database [47] (release 132). Taxonomic assignments were confirmed using blast analysis (blastn 2.11.0+ [48, 49]) against the NCBI nucleotide collection (nt) database (accessed January 2021). Taxonomic lineage was then retrieved from the NCBI taxonomy database using TaxonKit [50] and adjusted to the lowest common ancestor based on percentage identity and e-value score.

Tick 12S metabarcoding sequences generated were analysed via the USEARCH v11 [51] pipeline. Briefly paired-end reads were merged and sequences matching forward and reverse primers were retrieved (maximum number of mismatches=2). Sequences were then quality filtered and singletons were removed. The unoise3 [52] algorithm was used to perform denoising (error-correction) and generate zero-radius taxonomic units (zOTUs) equivalent to ASVs. Taxonomy was assigned using blast analysis and lineage was retrieved as outlined above for 16S metabarcoding.

Data visualization and statistical analysis was performed in RStudio (v1.4) with R version 4.0.2. The main R packages used were ampvis2 v2.6.7, phyloseq v1.34.0, microbiomeutilities v1.00.12 and vegan v2.5–7. The R package decontam v1.10.0 [53] was used to inspect data for cross-contamination and cross-talk between samples and to establish read cut-off thresholds (prevalence threshold=0.05). Identified contaminant ASVs (*n*=104), control samples (*n*=65) and sequence values <100 were removed. Rarefaction curves were generated using a step size of 100. Alpha diversity was measured using four indexes (number of observed ASVs, Chao1, Shannon and invSimpson) and statistical analysis was calculated using a Wilcoxon pairwise test (non-parametric) between sample types (blood, tick and tissue). Heatmaps were generated using ampvis2, where data were first aggregated to the family level and transformed to relative abundance.

Constrained ordination analysis was performed using two methods, canonical correspondence analysis and redundancy analysis (constrained principal component analysis), to investigate the relationships between sample types. Prior to the analysis, ASVs of <0.1 relative abundance and samples with <1000 sequences were removed. Data were first transformed using the Hellinger transformation and distance was measured using the Bray–Curtis method. Beta diversity statistical analysis was performed using ANOVA and permutational multivariate analysis of variance (PERMANOVA) (999 permutations) via the adonis function in the vegan package [54] (Hellinger transformation and Bray–Curtis distance measure); the F statistic and *P* value are presented (full statistical output available in Supplementary file 1). Hierarchical cluster analysis was performed using the euclidean distance measure (average), where data were first transformed using Hellinger transformation.

The composition for taxa of interest was aggregated to the family level and compared between the three sample types. Taxa of interest were defined as bacteria related to known pathogens associated with ticks and other vectors described globally, as outlined in Egan *et al*. [18]. Statistical analysis was performed using the Wilcoxon pairwise test (non-parametric). To identify shared ASVs (i.e. core microbiome) between sample types and species, data were transformed to relative abundance and assigned to the best taxonomic hit using the microbiomeutilities R package. Similarities of the core microbiome between the three sample types were investigated using thresholds of abundance and prevalence of 0.0001−0.001 and 0.001–0.05 respectively. For subsequent comparisons of the core microbiome between host and tick species, abundance and prevalence levels were set at 0.001 and 0.05, respectively. Prevalence data for taxa of interest are presented for samples based on 16S rRNA metabarcoding. Where ticks have been pooled, prevalence is reported based on minimum infection rates (i.e. assuming only one positive tick in each pool) [55].

Scripts for data analysis are available at https://github.com/siobhon-egan/wildlife-bacteria and https://github.com/siobhon-egan/wildlife-ticks, and raw Illumina MiSeq data have been deposited in the European Nucleotide Archive under project accession numbers PRJEB46056 (16S rRNA bacteria) and PRJEB46056 (12S rRNA ticks).

### Target PCRs

A nested PCR was used to amplify an ~1.4 kb fragment of 16S rRNA locus for *Neoehrlichia* and *Ehrlichia *. Amplicon PCRs were carried out in 25 μl reactions each containing: 1× buffer (KAPA Biosystems), 2.5 mM MgCl_2_, 0.4 μM of each primer, 0.25 mM of each dNTP, 0.5 U of Taq (KAPA Biosystems) and 2 μl of gDNA or 1 µl of primary product. Thermal cycling conditions were as follows: 95 °C for 3 min, followed by 40 cycles of 95 °C for 30 s, 48 °C (primary) or 54 °C (secondary) for 1 min, 72 °C for 2 min; and a final extension of 72 °C for 5 min.

For *Bartonella *, a PCR was used to amplify an ~370–450 bp fragment of the 16S rRNA – 23S internal transcribed spacer (ITS) region. Amplicon PCRs were carried out in 25 μl reactions each containing: 1× buffer (KAPA Biosystems), 2.0 mM MgCl_2_, 0.4 μM of each primer, 0.25 mM of each dNTP, 0.5 U of Taq (KAPA Biosystems) and 2 μl of gDNA. Thermal cycling conditions were as follows: 95 °C for 3 min, followed by 40 cycles of 95 °C for 15 s, 66 °C 15 s, 72 °C for 18 s; and a final extension of 72 °C for 5 min.

Amplicons were visualized on agarose gel and products of the expected size were excised with a sterile scalpel blade and purified using an in-house filtered pipette tip method [56]. Sanger sequencing was performed at the Australian Genome Research Facility (Perth, Western Australia) on an Applied Biosystems 3730xl DNA Analyzer using a BigDye Terminator v3.1 Cycle Sequencing Kit.

### Phylogeny

Nucleotide sequences were inspected and quality filtered using Geneious 10.2.6 (https://www.geneious.com). Identity was confirmed using blast analysis (blastn 2.11.0+ [48, 49]) against the NCBI nucleotide collection (nt) database. Sequences generated were aligned with references retrieved from GenBank [57] using muscle [58] (Anaplasmataceae and *Borrelia *) or clustal w [59] (* Bartonella *). The clustal w aligment method was identified as most suitable for *Bartonella* analysis due to the variable length of the 16S rRNA and ITS regions targeted. Phylogenies were inferred using the maximum-likelihood (ML) method. The optimal evolutionary model was selected using ModelFinder [60] based on the Bayesian information criterion. Phylogenetic analysis was performed in IQ-TREE v1.6.11 [61] and bootstrap support was calculated using the ultrafast (UFBoot2) method with 10000 replicates [62].

## Results

### Next-generation sequencing

The hosts species sampled in the present study were the black rat (*Rattus rattus*), brown antechinus (*Antechinus stuartii*), brush-tailed possum (*Trichosurus vulpecula*), bush rat (*Rattus fuscipes*), chuditch (*Dasyurus geoffroii*), Rusa deer (*Rusa timorensis*), long-nosed bandicoot (*Perameles nasuta*), quenda (*Isoodon fusciventer*), rabbit (*Oryctolagus cuniculus*) and swamp rat (*Rattus lutreolus*) (Table 1). Ticks identified from wildlife hosts were *Am. triguttatum*, *Ix. antechini*, *Ix. australiensis*, *Ix. holocyclus*, *Ix. tasmani* and *Ix. trichosuri*. Molecular screening was performed on 257 ticks pooled into 205 gDNA extracts (Table 3). Metabarcoding of ticks at the 12S rRNA locus confirmed morphological analysis and accurate differentiation of morphologically similar species. These results identified that mixed tick species were detected in five gDNA pools and, in all cases, these were larvae *of Ix. holocyclus* and *Ix. trichosuri*. A subset of representative 12S rRNA tick zOTU sequences were deposited under accession numbers MW665133–MW665150.

**Table 3.** Summary of tick species collected from wildlife; sample numbers in the table refer to the number of gDNA extracts with total number of tick specimens included in parentheses

Host	Tick species	Larvae	Nymph	Male	Female
Black rat	*Ix. holocyclus*	6 (12)	5 (5)	0	0
	*Ix. tasmani*	30 (38)	31 (34)	0	0
	*Ix. trichosuri*	4 (9)	2 (3)	0	0
	*Ix. holocyclus*; *Ix. trichosuri*	1 (3)	0	0	0
Brown antechinus	*Ix. antechini*	0	0	0	1 (1)
	*Ix. holocyclus*	1 (1)	0	0	0
	*Ix. tasmani*	2 (2)	2 (2)	0	0
Brush-tailed possum	*Am. triguttatum*	2 (3)	0	0	0
	*Ix. holocyclus*	0	3 (3)	0	5 (5)
	*Ix. tasmani*	0	0	0	2 (2)
	*Ix. trichosuri*	1 (1)	2 (2)	0	18 (18)
Bush rat	*Ix. tasmani*	1 (1)	3 (3)	0	0
Chuditch	*Am. triguttatum*	1 (1)	0	0	0
Long-nosed bandicoot	*Ix. holocyclus*	8 (9)	5 (5)	5 (5)	20 (20)
	*Ix. tasmani*	5 (7)	12 (12)	0	0
	*Ix. trichosuri*	3 (3)	2 (2)	0	0
Quenda	*Am. triguttatum*	0	1 (1)	0	0
	*Ix. australiensis*	0	1 (1)	0	0
Rabbit	*Ix. holocyclus*	3 (6)	6 (10)	0	0
	*Ix. tasmani*	0	2 (2)	0	0
	*Ix. trichosuri*	1 (4)	4 (6)	0	0
	*Ix. holocyclus*; *Ix. trichosuri*	3 (12)	1 (3)	0	0

### Bacteria 16S metabarcoding

A total of 536 gDNA extracts from 203 individual animals (159 blood, 205 tick pools, 172 tissue samples) underwent bacterial profiling. An additional 65 control samples (EXB and NTC) were also sequenced to obtain background laboratory and reagent profiles. Bacterial 16S metabarcoding produced 102,503, 643 raw sequences (Table S1, Fig. S1; samples=96,969,355, controls=5,534,288). Blood samples produced 35,160,160 sequences (mean=185 053), tick samples produced 24,844, 688 sequences (mean=95,556) and tissue samples produced 36,964, 507 sequences (mean=197, 671). Following quality filtering, denoising and merging, a total of 64, 186,307 sequences (66.2 %) were obtained from the samples; the mean for blood samples was 121 376 (65.6 %), mean for tick samples 70, 650 (73.9 %) and mean for tissue samples 121,689 (61.6 %).

**Fig. 2.**
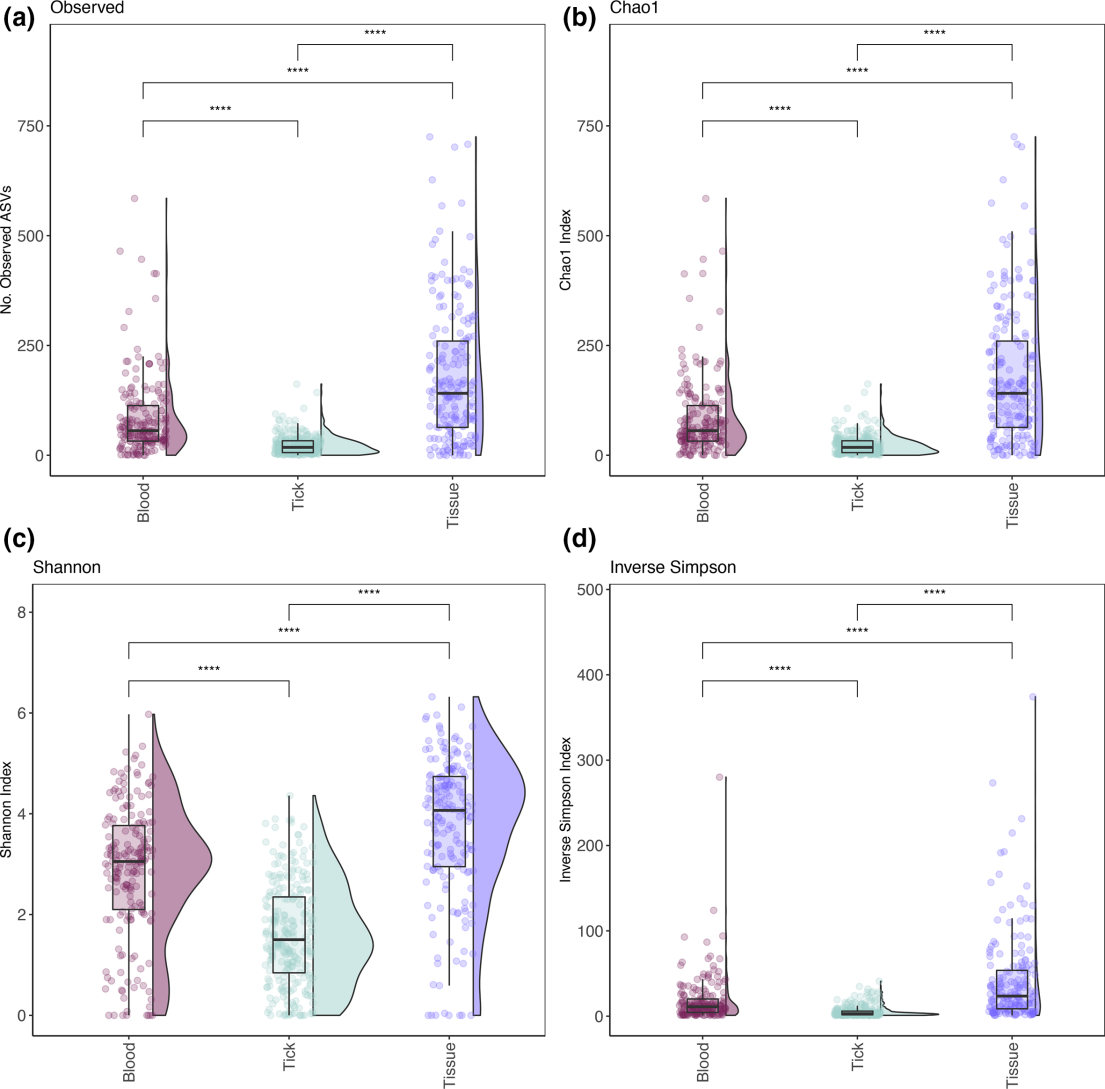
Boxplot of Alpha-diversity indices. Diversity indexes: (**a**) observed number of ASVs, (**b**) Chao1 index, (**c**) Shannon index and (**d**) inverse Simpson index. Boxplots and violin plots represent the distribution of diversity among samples within their category: blood (159), tick (205 pools) and tissue (172). Statistical analysis between sample types was done using Wilcoxon pairwise (non-parametric) test with significance values indicated as follows: NS for *P*>0.05; * for *P*≤0.05; ** for *P*≤0.01; *** for *P*≤0.001; **** for *P*≤0.0001.

After contaminating taxa and controls were removed, a total of 31,194 bacterial ASVs were identified and 5243 ASVs had an abundance of over 1000 sequences. Rarefaction curves (Fig. S2) showed the number of ASVs generally plateaued at a depth of 60, 000, 40,000 and 50,000 sequences for blood, tick and tissue samples, respectively. Alpha diversity analysis (Fig. 2) identified significant variation between sample types (Wilcoxon pairwise test, *P*<0.001). The highest number of observed ASVs was in tissue samples (21, 811), followed by blood (9,401) and ticks (3,568). Tissue samples showed the highest level of alpha diversity across all measures of alpha diversity. *Ixodes australiensis* and *Ix. trichosuri* had the highest alpha diversity of the tick species sampled (Fig. S3). Once identified, contaminant taxa and controls were removed and the dominant phyla identified were *Proteobacteria* (37.3 %), *Firmicutes* (27.8 %), *Actinobacteria* (14.7 %) and *Bacteroidetes* (10.7 %) (Figs S4 and S5).

**Fig. 3.**
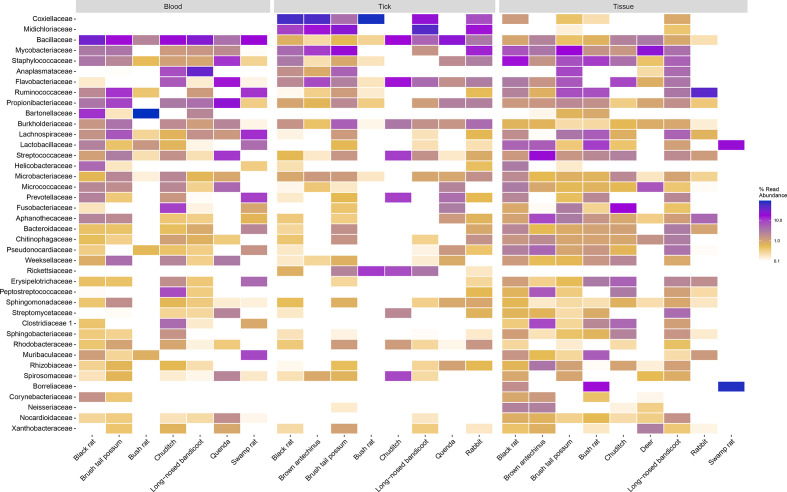
Heatmap of the top 40 most prevalent bacterial family taxa identified in wildlife blood, tick and tissue samples. Data were first transformed to relative sequence abundance.

**Fig. 4.**
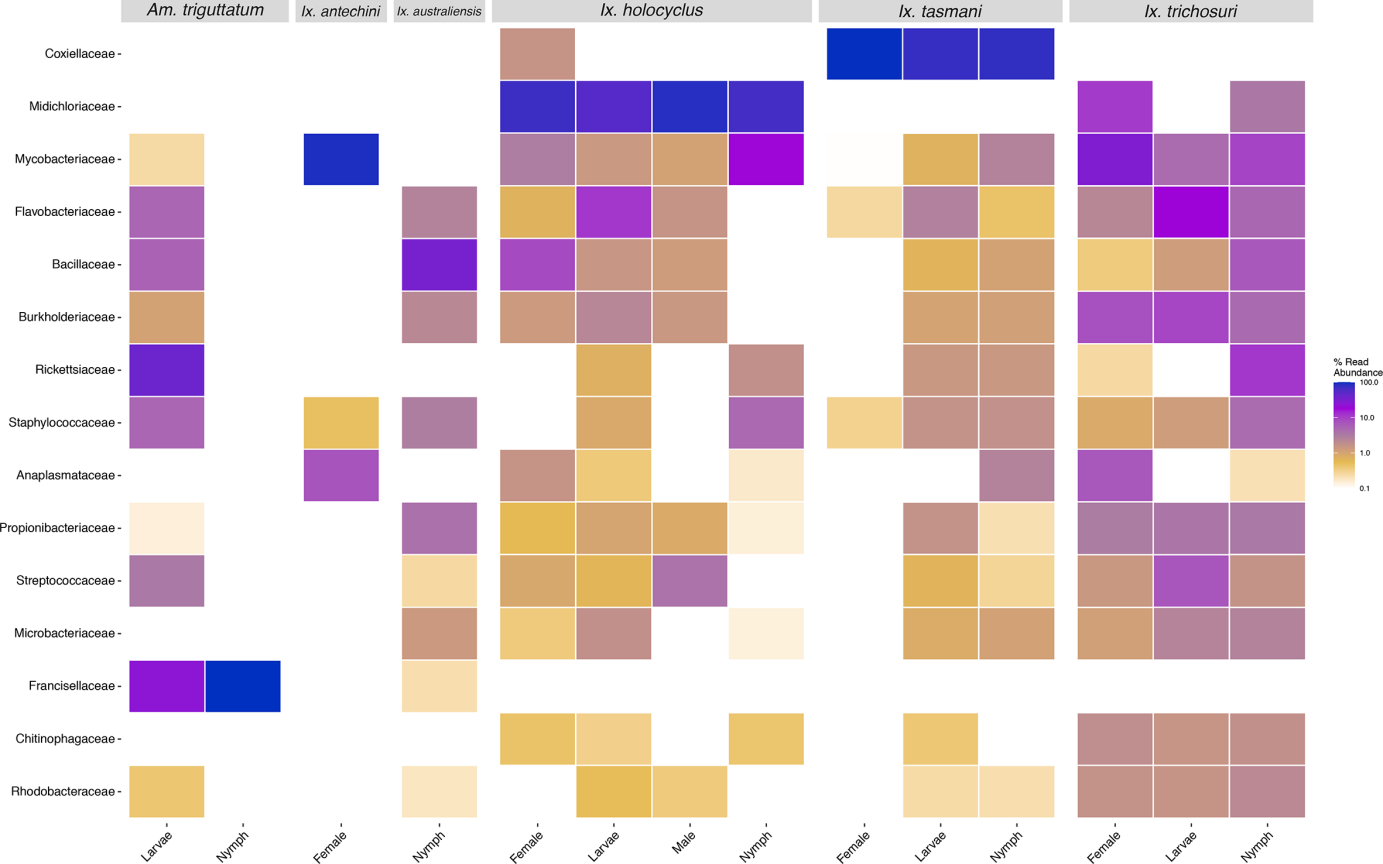
Heatmap of the top 15 most prevalent bacterial family taxa identified in tick samples, showing abundance in species and instar. Data were first transformed to relative sequence abundance. Tick samples consisting of mixed species pools were excluded.

In blood samples, the most abundant families were *Bacillaceae* (22.9 %), *Bartonellaceae* (11.3 %), *Anaplasmataceae* (7.3 %) and *Mycobacteriaceae* (2.6 %) (Fig. S6). In tick samples, the most abundant families were *Coxiellaceae* (33.8 %), *Midichloriaceae* (31.0 %), *Mycobacteriaceae* (6.0 %), *Flavobacteriaceae* (2.6 %) and *Anaplasmataceae* (1.8 %) (Fig. S7). In tissue samples, the most abundant families were *Staphylococcaceae* (8.2 %), *Mycobacteriaceae* (6.5 %), *Ruminococcaceae* (5.5 %), *Flavobacteriaceae* (4.7 %) and *Anaplasmataceae* (2.7 %) (Fig. S8). Comparison of family taxa showed differences in abundance among sample types and species (and tick instar) (Figs. 3 and 4).

**Fig. 5.**
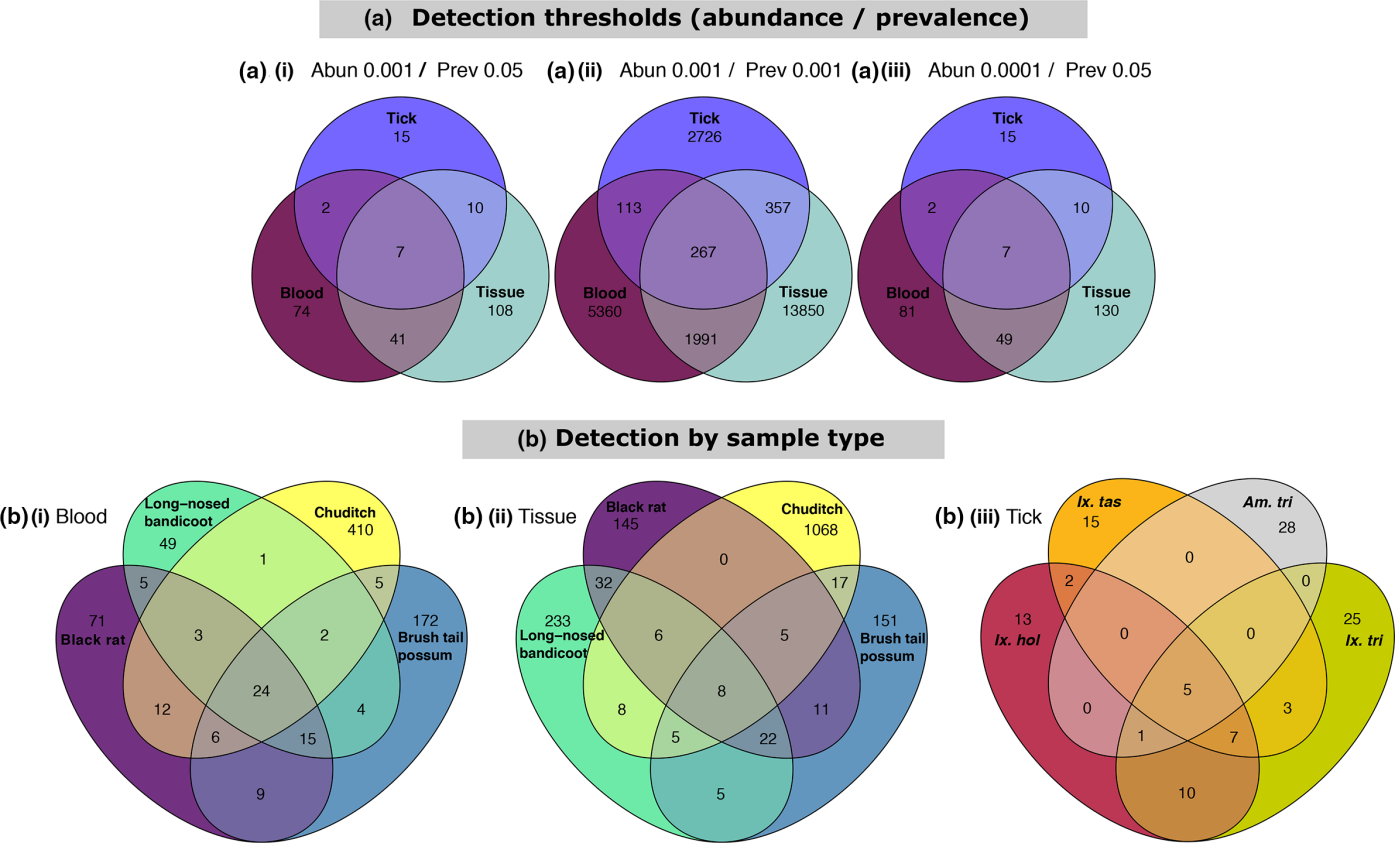
Venn diagram of core taxa present within samples presented as detection of ASVs using abundance and prevalence. Data were first transformed to relative abundance and ASVs assigned to the best hit. (**a**) Impact of similarity based on detection thresholds of abundance and prevalence as (**i**) 0.001 and 0.05, (ii) 0.001 and 0.001, and (iii) 0.0001 and 0.05. (**b**) Core taxa present within sample types using detection levels set at 0.001 and 0.05 for abundance and prevalence, respectively, for (**i**) blood, (ii) tissue and (iii) ticks. For within-sample comparisons, only the four most abundant vertebrate host/tick species were selected. Tick species abbreviations: *Amblyomma triguttatum* (Am. tri), *Ixodes holocyclus* (Ix. hol), *Ixodes tasmani* (Ix. tas), *Ixodes trichosuri* (Ix. tri). Tick samples consisting of mixed species pools were excluded.

The overlap of samples at the ASV level was investigated using various thresholds to define the ‘core microbiome’, with two out of three models showing only seven shared ASVs between all three sample types (Fig. 5). Shared core microbiome ASVs of blood and tissue samples were compared between the four most abundant host samples and tick species (Fig. 5).

**Fig. 6.**
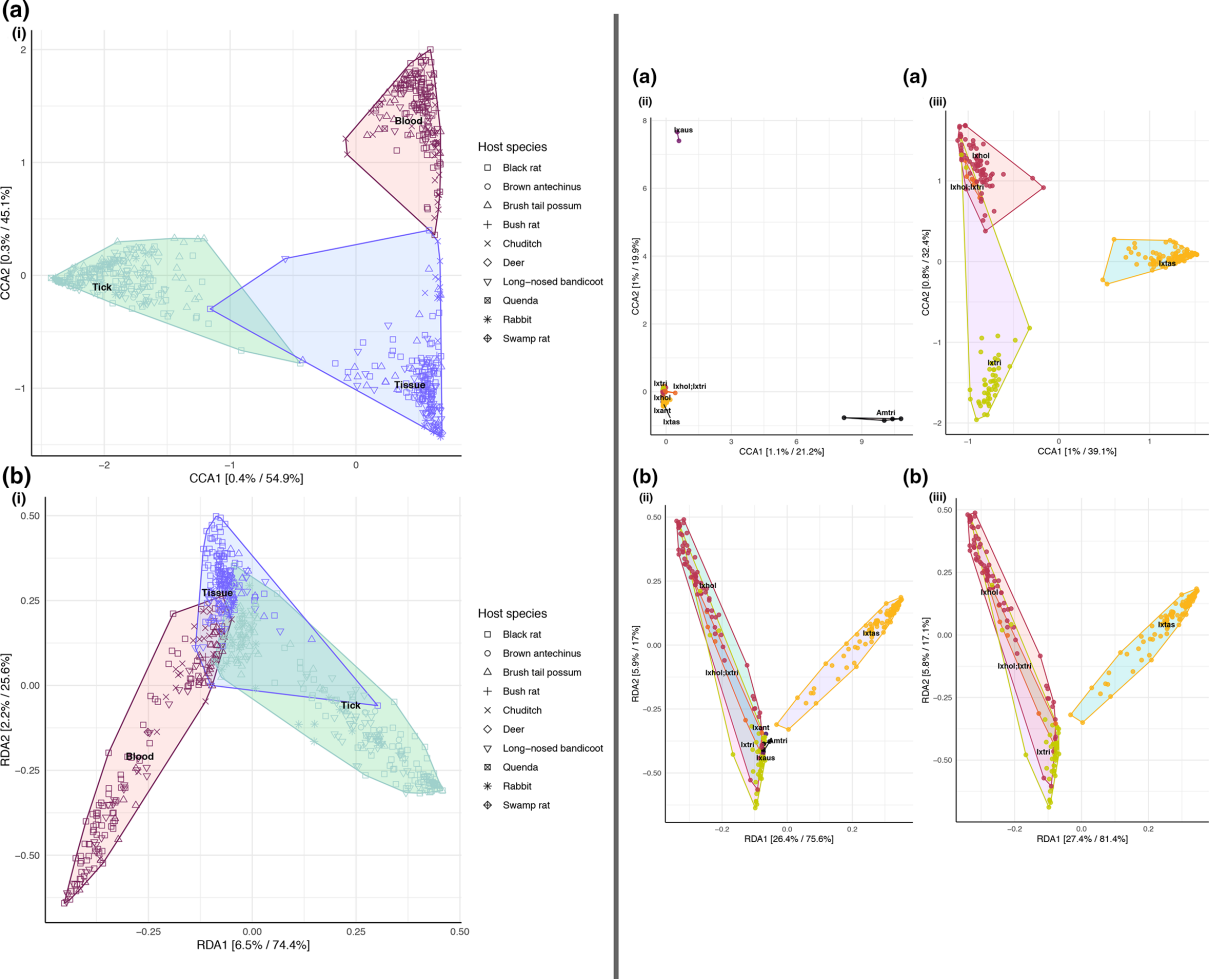
Constrained ordination plot of microbiome composition from using constrained methods: (**a**) canonical correspondence analysis and (**b**) redundancy analysis (constrained principal component analysis). (**i**) Ordination analysis of all samples, *N*=536 (159 blood, 205 tick pools and 172 tissue) by sample type. (ii) Ordination of all tick samples by tick species. (iii) Ordination of three co-habiting tick species in Sydney Northern Beaches area. Prior to the analysis, ASVs with <0.1 relative abundance were removed. Analysis was done using the Hellinger transformation and Bray–Curtis distance measure. The relative contribution (eigen value) of each axis to the total inertia in the data as well as to the constrained space only, respectively, are indicated in percent on the axis titles. Tick species abbreviations: *Amblyomma triguttatum* (Am. tri), *Ixodes antechinus* (Ix. ant), *Ixodes australiensis* (Ix. aus), *Ixodes holocyclus* (Ix. hol), *Ixodes tasmani* (Ix. tas), *Ixodes trichosuri* (Ix. tri).

Ordination analysis revealed a distinct difference between the bacterial composition of blood, tick and tissue samples (Fig. 6). Hierarchical cluster analysis (Fig. S9) and statistical analysis (PERMANOVA *F*=36.209, *P*=0.001, see Supplementary file S1) also supported this finding showing that there was a statistical difference in sample types, and there was little overlap in the microbiome composition of blood and tick samples. Tissue samples were identified as an intermediate sample type and showed some (albeit low) similarities to blood and tick samples. Ordination analysis of tick samples showed distinction between tick species (Fig. 6). The tick species *Ix. holocyclus* and *Ix. trichosuri* showed the most similar bacterial composition with ordination analysis demonstrating overlap between these two species (PERMANOVA, *F*=14.01, *P*=0.001, see Supplementary file S1). Although sampling numbers were uneven among host species, which limited statistical inference, hierarchical cluster analysis did show that samples grouped were based on host (and tick) species (Fig. S10).

### Taxa of interest

**Fig. 7.**
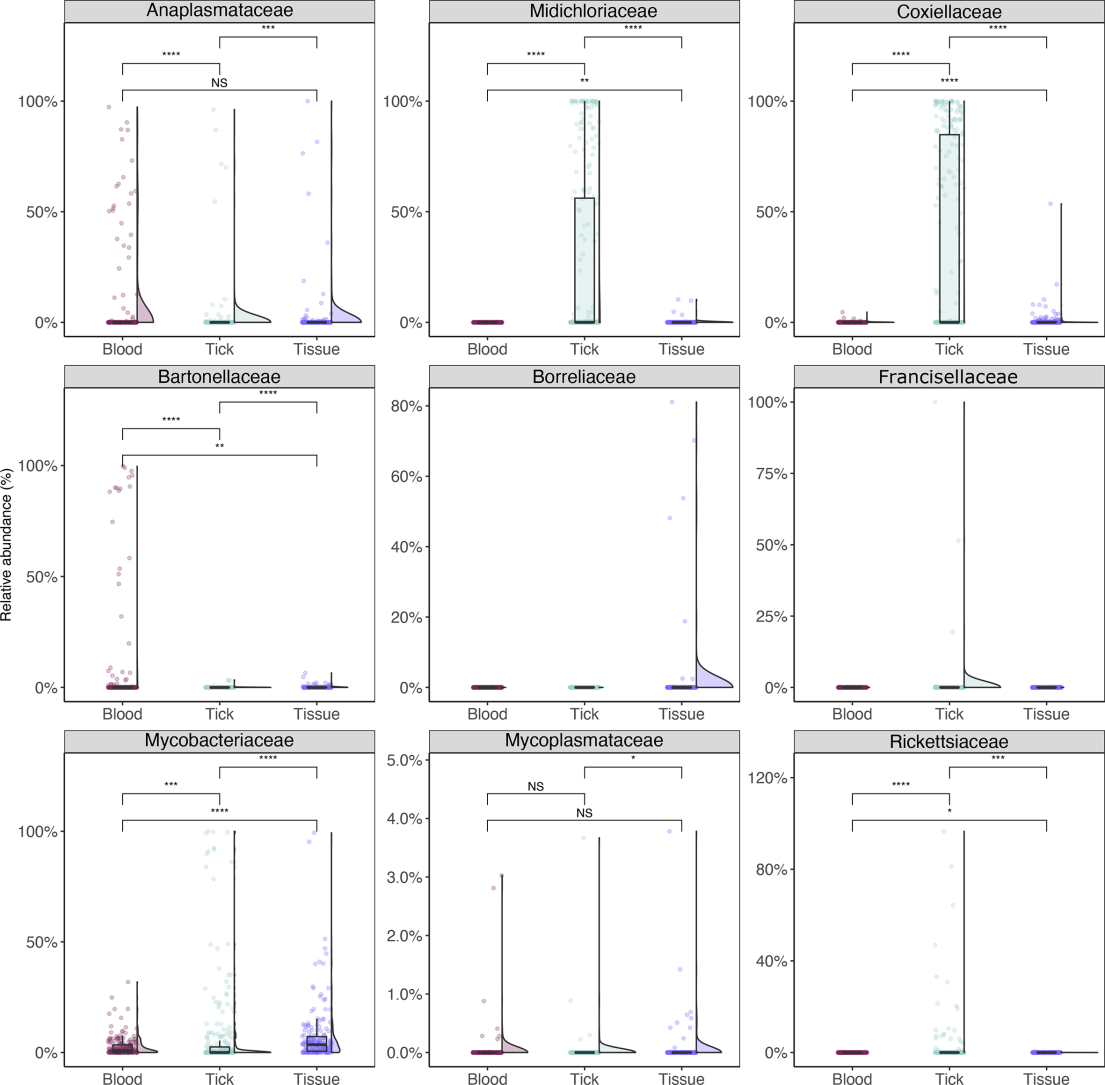
Relative abundance for select bacterial taxa of interest (aggregated to the family level) from wildlife samples. Statistical analysis between sample types was done using a Wilcoxon pairwise (non-parametric) test with significance values indicated as follows: NS for *P*>0.05; * for *P*≤0.05; ** for *P*≤0.01; *** for *P*≤0.001; **** for *P*≤0.0001. Taxa of interest are defined as bacteria related to known pathogens associated with ticks and other vectors described globally, as outlined in Egan *et al*. [18].

Nine family taxa were chosen for comparison among sample types (Fig. 7). *Anaplasmataceae* bacteria were most prevalent in blood samples (both by relative abundance and prevalence). Blood and tissue samples showed no statistical difference in relative abundance (Wilcoxon pairwise test, *P*=0.26), with significantly lower abundance in tick samples (Wilcoxon pairwise test, *P*<0.001). There was weak support for differences in *Mycoplasmataceae* between sample types (Wilcoxon pairwise test, *P*=0.023–0.43). *Midichloriaceae *, *Coxielleaceae* and *Rickettsiaceae* were all significantly more abundant in ticks than in blood or tissue samples. Statistical comparisons of *Borreliaceae* and *Francisellaceae* were not made due to the small number of positive samples.

For subsequent prevalence analysis only taxa of interest that could be accurately defined to the genus level were retained (as per blast results). This identified 235 ASVs from 364 samples and 159 individuals (see Table 4 and full sequence data and blast results in Supplementary file S2).

**Table 4.** Presence of taxa of interest* by bacteria metabarcoding Prevalence is reported as the proportion of individual hosts positive with the proportion of samples positive in parentheses (accounting for instances where more than one sample type was collected from an individual).

Taxa of interest	Sample type	Black rat	Brown antechinus	Brush tail possum	Bush rat	Chuditch	Deer	Long-nosed bandicoot	Quenda	Rabbit	Swamp rat
* Anaplasmataceae *	Blood	4/68 (4/84)	0/0 (0/0)	5/18 (6/21)	0/2 (0/2)	7/22 (9/24)	0/0 (0/0)	18/20 (22/25)	0/1 (0/2)	0/0 (0/0)	0/1 (0/1)
	Tick	3/54 (3/79)	1/4 (1/6)	4/15 (5/33)	0/3 (0/4)	0/1 (0/1)	0/0 (0/0)	8/31 (9/60)	0/2 (0/2)	0/7 (0/20)	0/0 (0/0)
	Tissue	6/71 (8/80)	0/2 (0/2)	4/16 (5/20)	0/3 (0/3)	1/9 (1/9)	3/3 (3/6)	18/40 (19/44)	0/0 (0/0)	0/7 (0/7)	0/1 (0/1)
* Bartonella *	Blood	22/68 (27/84)	0/0 (0/0)	7/18 (8/21)	2/2 (2/2)	0/22 (0/24)	0/0 (0/0)	4/20 (4/25)	0/1 (0/2)	0/0 (0/0)	0/1 (0/1)
	Tick	3/54 (4/79)	0/4 (0/6)	0/15 (0/33)	1/3 (1/4)	0/1 (0/1)	0/0 (0/0)	1/31 (1/60)	0/2 (0/2)	0/7 (0/20)	0/0 (0/0)
	Tissue	4/71 (5/80)	1/2 (1/2)	9/16 (11/20)	2/3 (2/3)	0/9(0/9)	0/3 (0/6)	3/40 (3/44)	0/0 (0/0)	0/7 (0/7)	0/1 (0/1)
* Borrelia *	Blood	0/68 (0/84)	0/0 (0/0)	0/18 (0/21)	0/2 (0/2)	0/22 (0/24)	0/0 (0/0)	0/20 (0/25)	0/1 (0/2)	0/0 (0/0)	0/1 (0/1)
	Tick	0/54 (0/79)	0/4 (0/6)	0/15 (0/33)	0/3 (0/4)	0/1 (0/1)	0/0 (0/0)	0/31 (0/60)	0/2 (0/2)	0/7 (0/20)	0/0 (0/0)
	Tissue	7/71 (7/80)	0/2 (0/2)	0/16 (0/20)	1/3 (1/3)	0/9 (0/9)	0/3 (0/6)	0/40 (0/44)	0/0 (0/0)	0/7 (0/7)	1/1 (1/1)
* Coxiellaceae *	Blood	7/68 (7/84)	0/0 (0/0)	1/18 (1/21)	0/2 (0/2)	0/22 (0/24)	0/0 (0/0)	3/20 (5/25)	0/1 (0/2)	0/0 (0/0)	0/1 (0/1)
	Tick	50/54 (63/79)	3/4 (4/6)	8/15 (9/33)	3/3 (4/4)	0/1 (0/1)	0/0 (0/0)	22/31 (24/60)	0/2 (0/2)	2/7 (2/20)	0/0 (0/0)
	Tissue	33/71 (38/80)	0/2 (0/2)	2/16 (2/20)	2/3 (2/3)	0/9 (0/9)	0/3 (0/6)	20/40 (22/44)	0/0 (0/0)	0/7 (0/7)	0/1 (0/1)
* Francisellaceae *	Blood	0/68 (0/84)	0/0 (0/0)	0/18 (0/21)	0/2 (0/2)	0/22 (0/24)	0/0 (0/0)	0/20 (0/25)	0/1 (0/2)	0/0 (0/0)	0/1 (0/1)
	Tick	0/54 (0/79)	0/4 (0/6)	2/15 (2/33)	0/3 (0/4)	0/1 (0/1)	0/0 (0/0)	0/31 (0/60)	2/2 (2/2)	0/7 (0/20)	0/0 (0/0)
	Tissue	0/71(0/80)	0/2(0/2)	0/16 (0/20)	0/3 (0/3)	0/9 (0/9)	0/3 (0/6)	0/40 (0/44)	0/0 (0/0)	0/7 (0/7)	0/1 (0/1)
* Midichloria *	Blood	0/68 (0/84)	0/0 (0/0)	0/18 (0/21)	0/2 (0/2)	0/22 (0/24)	0/0 (0/0)	0/20 (0/25)	0/1 (0/2)	0/0 (0/0)	0/1 (0/1)
	Tick	17/54 (21/79)	2/4 (2/6)	12/15 (20/33)	0/3 (0/4)	0/1 (0/1)	0/0 (0/0)	19/31 (41/60)	0/2 (0/2)	5/7 (10/20)	0/0 (0/0)
	Tissue	5/71 (5/80)	0/2 (0/2)	1/16 (1/20)	0/3(0/3)	0/9 (0/9)	0/3 (0/6)	3/40 (3/44)	0/0 (0/0)	0/7 (0/7)	0/1 (0/1)
* Mycoplasma *	Blood	1/68 (1/84)	0/0 (0/0)	0/18 (0/21)	0/2(0/2)	0/22 (0/24)	0/0 (0/0)	0/20 (0/25)	0/1 (0/2)	0/0 (0/0)	1/1 (1/1)
	Tick	0/54 (0/79)	0/4 (0/6)	0/15 (0/33)	0/3 (0/4)	0/1 (0/1)	0/0 (0/0)	0/31 (0/60)	0/2 (0/2)	0/7 (0/20)	0/0 (0/0)
	Tissue	1/71 (1/80)	0/2 (0/2)	0/16 (0/20)	0/3 (0/3)	0/9 (0/9)	0/3 (0/6)	0/40 (0/44)	0/0 (0/0)	0/7 (0/7)	0/1 (0/1)
* Rickettsia *	Blood	0/68 (0/84)	0/0 (0/0)	0/18 (0/21)	0/2 (0/2)	0/22 (0/24)	0/0 (0/0)	0/20 (0/25)	0/1 (0/2)	0/0 (0/0)	0/1 (0/1)
	Tick	7/54 (7/79)	0/4 (0/6)	2/15 (2/33)	2/3 (3/4)	1/1 (1/1)	0/0 (0/0)	9/31 (10/60)	0/2 (0/2)	1/7 (1/20)	0/0 (0/0)
	Tissue	0/71 (0/80)	0/2 (0/2)	0/16 (0/20)	0/3 (0/3)	0/9 (0/9)	0/3 (0/6)	0/40 (0/44)	0/0 (0/0)	0/7 (0/7)	0/1 (0/1)

*Taxa of interest are defined as bacteria related to known pathogens associated with ticks and other vectors described globally, as outlined in Egan *et al.* [18].

#### Anaplasmataceae

The family *Anaplasmataceae* was identified in 95 samples from 59 individuals. The most prevalent genera were *Neoehrlichia* (43 individuals), followed by *Ehrlichia* (eight individuals) and *Anaplasma* (three individuals).

‘*Candidatus* Neoehrlichia arcana’ was identified in 52 samples: 29 blood, seven ticks (*Ix. holocyclus*) and 16 tissue. ‘*Candidatus* Neoehrlichia australis’ was identified in 33 samples: eight blood, six ticks (*Ix. holocyclus*, *Ix. trichosuri*) and 19 tissue. Sixteen samples had mixed infections of ‘*Ca*. N. arcana’ and ‘*Ca*. N. australis’ (six blood and ten tissue). No ticks had mixed *Neoehrlichia* infections. Three putative novel *Anaplasmataceae* species were identified from the present study, two *Ehrlichia* spp. and a single *Neoehrlichia* sp. The novel *Neoehrlichia* sp. was identified in three samples: a tissue sample from a black rat and a corresponding tick from the same individual (*Ix. tasmani*); and a tick from a brown antechinus (*Ix. antechini*). One novel *Ehrlichia* sp. was identified in five samples from two brush-tailed possums: two blood, one tick (*Ix. holocyclus*) and two tissue. A second novel *Ehrlichia* sp. was identified in seven samples from six chuditch (six blood and one tissue). Only one chuditch was positive in corresponding tissue and blood samples. Three deer tissue samples were infected with *Anaplasma platys* (Supplementary file S2). Detection of *Anaplasmataceae* bacteria differed between tick species and instars. *Ixodes holocyclus* females had the highest prevalence of this taxon, followed by nymph and larval ticks, while no males were positive. Only *Ix. tasmani* nymph samples were positive and in the case of *Ix. trichosuri*, females and nymphs were identified with *Anaplasmataceae* and all larvae were negative.

**Fig. 8.**
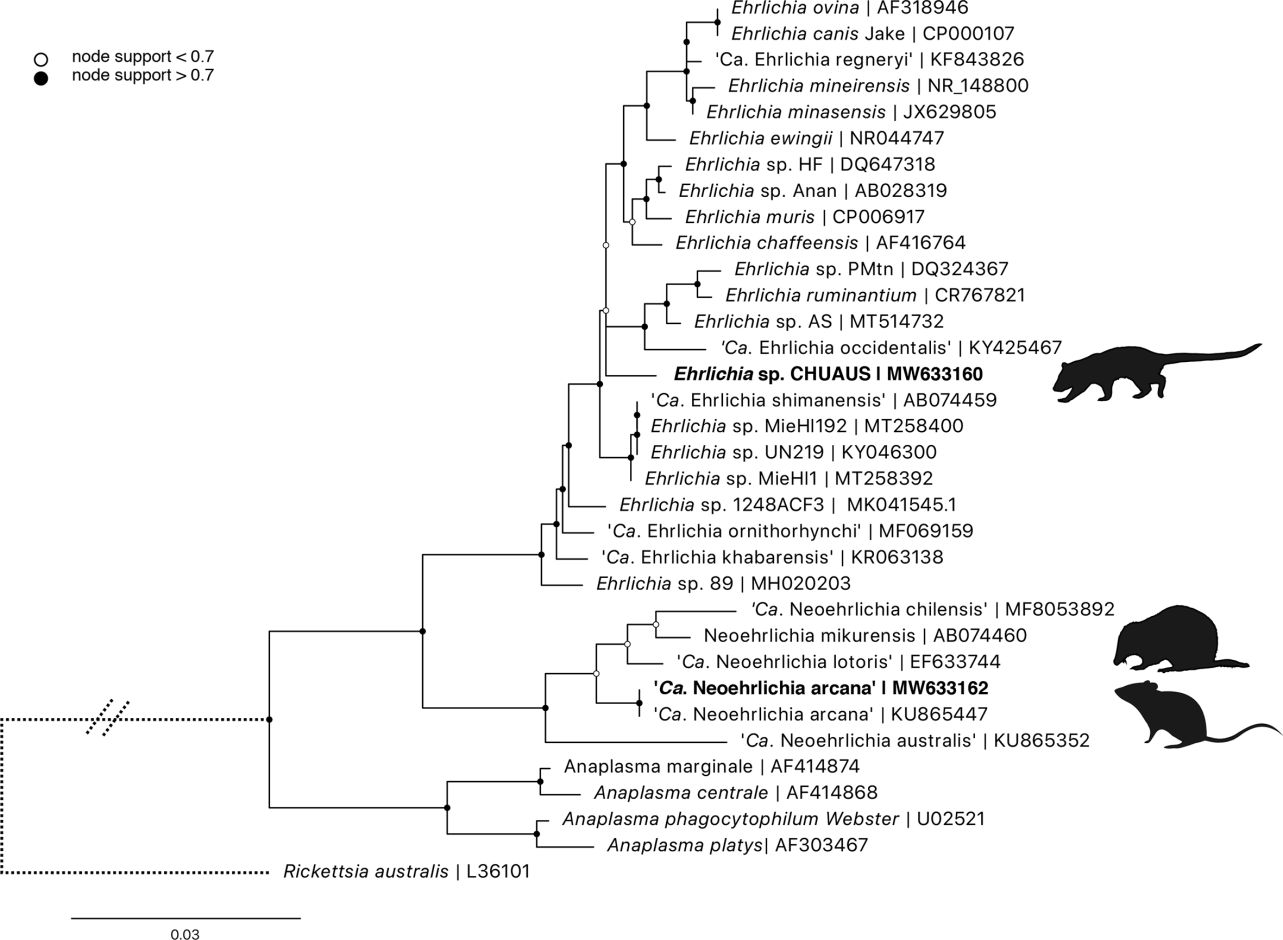
ML phylogenetic reconstruction of *Anaplasmataceae* based on a 1244 bp alignment of the 16S rRNA locus. Substitution model: K80+F with 10000 replicates. Node values correspond to bootstrap support where values >0.7 are indicated by shaded circles. Bar, number of substitutions per nucleotide position. Sequences generated in the present study are in bold type.

Phylogenetic analysis was performed using a 1,244 bp fragment of the 16S rRNA locus (Fig. 8). All ‘*Ca*. N. arcana’ amplified were identical to each other and showed 100% similarity to ‘*Ca*. Neoehrlichia arcana’ isolate HT94 from *Ix. holocyclus*, Australia (KU865447). *Ehrlichia* sequences from chuditch blood samples were 100% identical to each other and most similar to *Ehrlichia* sp. Anan from *Ix. ovatus*, Japan (AB028319, 98.94% similarity). The nearest named species was *Ehrlichia muris* (CP006917, 98.86% similarity) isolated from a wild mouse (*Eothenomys kageus*) in Japan (genetic distances in Supplementary file S3).

#### Midichloria

A total of 103 samples were positive for *Midichloria *, which were identified in 94 tick samples from 62 individual animals. Positive tick species included *Ix. holocyclus*, *Ix. tasmani* and *Ix. trichosuri* from black rats, brown antechinuses, brush-tailed possums, long-nosed bandicoots and rabbits from New South Wales. While *Midichloria* was identified in 13 *Ix. tasmani*, the relative abundance was low (108–363 sequences; 0.1–0.4% relative abundance). Nine tissue samples (nine individuals) were positive for *Midichloria *, but the number of sequences was generally low (111–14,002) when compared to tick samples; hosts included black rats (five), a brush-tailed possum (one) and long-nosed bandicoots (two). Only two individuals had positive tissue and tick samples, one brush-tailed possum and one long-nosed bandicoot. *Midichloria* was identified in all instars of *Ix. holocyclus* but only female and nymph stages of *Ix. trichosuri* (Fig. 4). No blood samples were positive for *Midichloria* (Fig. 3).

#### Coxiellaceae

The family *Coxiellaceae* was identified in 183 samples from 113 individuals. Eleven individuals had positive blood samples, although there was a relatively low number of sequences (100–2819), from hosts including the black rat (seven), brush-tailed possum (one) and long-nosed bandicoot (three). In total, 106 ticks from 88 individuals were positive: *Am triguttatum* (one), *Ix. tasmani* (90) *Ix. holocyclus* (nine), and *Ix. trichosuri*. Fifty-seven individuals were positive for *Coxiellaceae* in tissue samples, including the black rat (33), brush-tailed possum (two), bush rat (two) and long-nosed bandicoot (20). Forty-five individuals had at least two sample types positive for *Coxiellaceae *, and only two black rats had positive blood, tick and tissue samples. All instars of *Ix. tasmani* sampled (female, nymph, larva) were positive for *Coxiellaceae *, but only female *Ix. holocyclus* were positive.

#### Rickettsiaceae

*Rickettsia* was identified exclusively in 24 tick samples from 22 individuals. Positive samples from Western Australia were all identified as originating in *Am. triguttatum* (three) from brush-tailed possums and quenda. Ticks identified as positive from New South Wales were: *Ix. holocyclus* (nine) from black rats and long-nosed bandicoots, *Ix. tasmani* (15) from black rats, bush rats, long-nosed bandicoots and rabbits, and *Ix. trichosuri* (three) from long-nosed bandicoots. No tissue or blood samples were positive for *Rickettsia *.

#### Borrelia

A novel *Borrelia* sp. was identified in nine tissue samples: seven black rats, one bush rat and one swamp rat. The identity of these *Borrelia* sequences showed they were most similar to *Borrelia* R57 (AY626138, 95.68–96.70% similarity, Supplementary file S2). Corresponding blood and ticks from these individuals were negative and no other *Borrelia* sequences were identified from any other samples.

Attempts to amplify and perform Sanger sequencing on the *flaB* gene using pan-* Borrelia* primers [63] were unsuccessful. Given these failed attempts and the small volume of gDNA from samples (tissue biopsy punch only 2 mm), an alternative approach was employed to amplify additional fragments of the 16S rRNA v3–4 hypervariable region on the Illumina MiSeq.

**Fig. 9.**
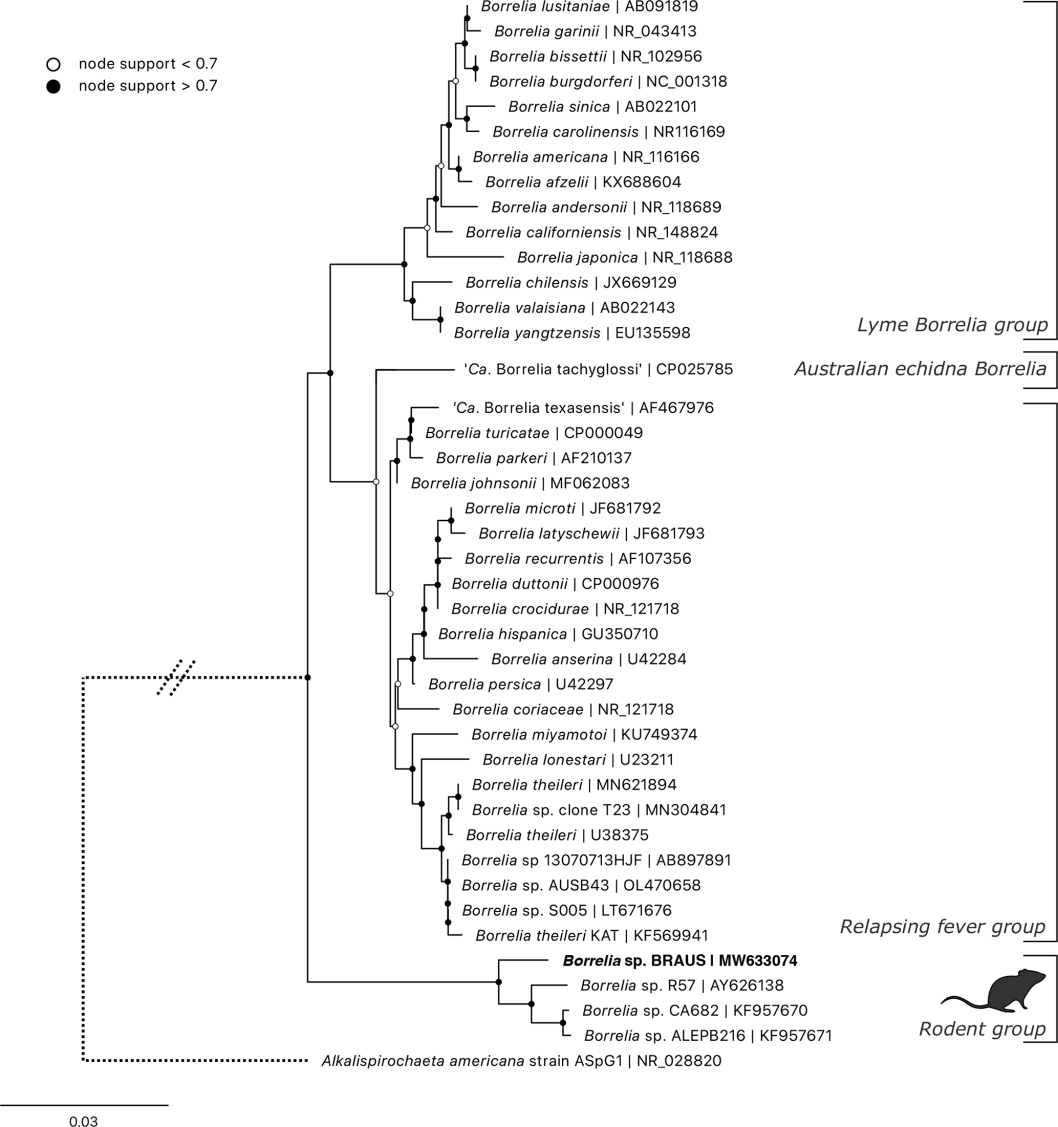
ML phylogenetic reconstruction of *Borrelia* based on a 431 bp alignment of the 16S rRNA locus (hypervariable region 3–4). Substitution model: K2P+G4 with 10000 replicates. Node values correspond to bootstrap support where values >0.7 are indicated by shaded circles. Bar, number of substitutions per nucleotide position. The sequence generated in the present study is shown in bold type.

After bioinformatic analysis outlined above, a single ASV was generated from all nine samples. Phylogeny was reconstructed using the ML method (GTR+R model) based on a 426 bp alignment of the 16S rRNA locus (hypervariable region v3–4) (Fig. 9). The novel *Borrelia* sequence showed 98.4% similarity to *Borrelia* sp. ALEPB216 (KF957671) from *R. rattus* in California, USA (genetic distances in Supplementary file S4). The next most similar sequences were *Borrelia* sp. CA682 (KF957670, similarity 98.1%) and *Borrelia* sp. R57 (AY626138, similarity 97.6%). The topology showed that these sequences formed a distinct ‘rodent’ clade, basal to the three major groups currently described (Fig. 9). The nearest named species was *Borrelia theileri* (U38375, similarity 94.50%).

#### Bartonella

*Bartonella* sp. was identified in 69 samples from 47 individuals. Blood samples represented the majority of *Bartonella* positives with 35 individuals, which included black rats (22), brush-tailed possums (seven), bush rats (two) and long-nosed bandicoots (four). Nineteen individuals had positive tissue samples: black rats (four), brown antechinus (one), brush-tailed possums (nine), bush rats (two) and long-nosed bandicoots (three); seven individuals had positive tissue and blood samples. Six tick samples were positive for *Bartonella* (from five individuals) with three tick species (*Ix. holocyclus*, *Ix. tasmani* and *Ix. trichosuri*) from black rats and long-nosed bandicoots. All tick samples that were positive also had corresponding positive blood or tissue samples.

**Fig. 10.**
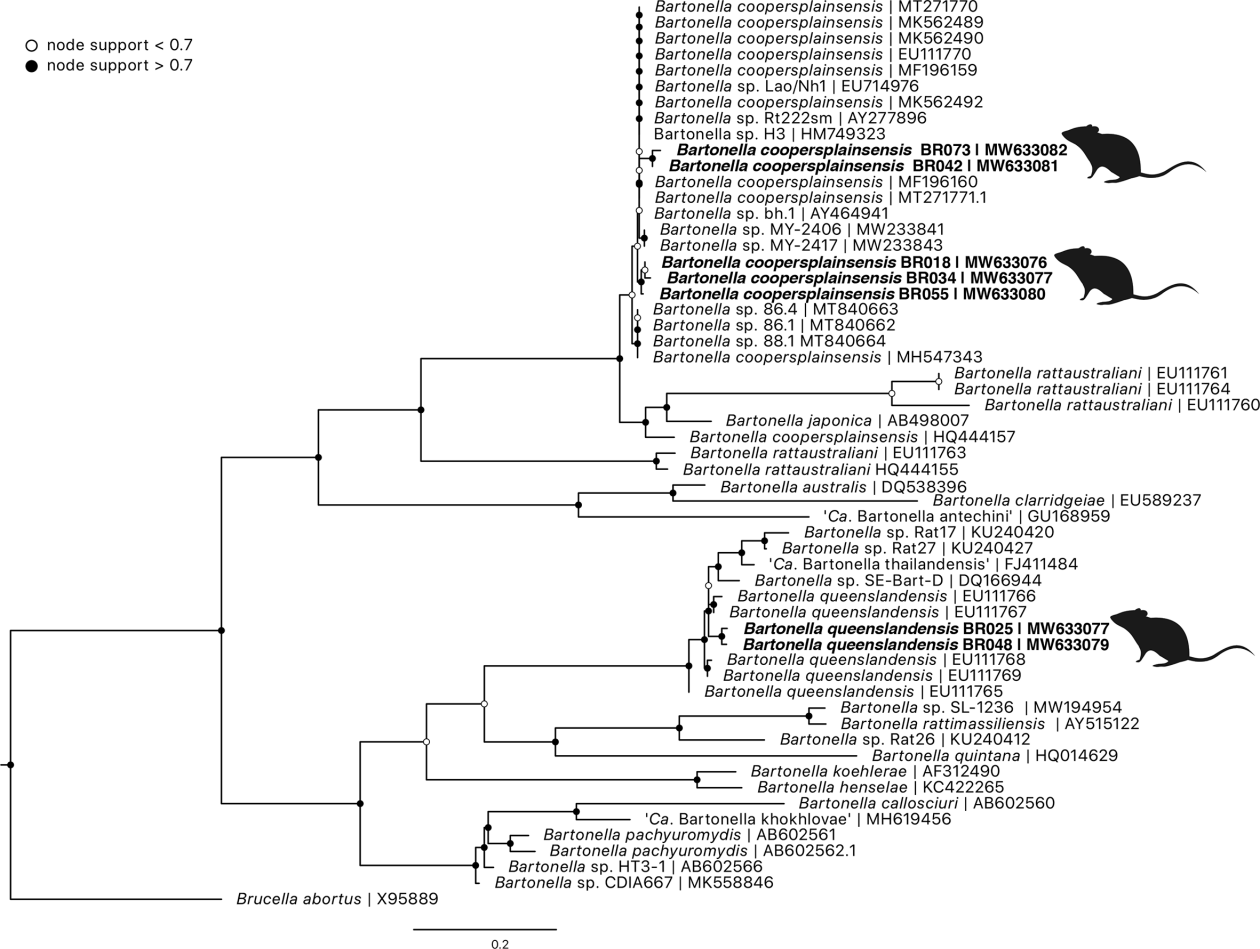
ML phylogenetic reconstruction of *Bartonella* based on a 533 bp alignment of the 16S–23S ITS region. Substitution model: HKY+F with 10000 replicates. Node values correspond to bootstrap support, where values >0.7 are indicated by shaded circles. Bar, number of substitutions per nucleotide position. Sequences generated in the present study are in bold type. Taxa of interest are defined as bacteria related to known pathogens associated with ticks and other vectors described globally, as outlined in Egan *et al*. [18].

Targeted sequencing on a subset of positive samples showed two genotypes of *Bartonella queenslandensis* that were 97.2% similar to each other with ten SNPs (genetic distances in Supplementary file S5). The next most similar sequence to genotype BR025 and BR048 was *Ba. queenslandensis* strain AUST/NH8 (EU111767) at 96.1 and 94.8% similarity, respectively. Comparison of *Ba. queenslandensis* type sequences showed divergence of up to 7.2% (between EU111765 and EU111766) within the species. Five genotypes of *Ba. coopersplainsensis* were identified with 94.4–99.2% similarity to each other, and 96.7–98.7% similarity to the *Ba. coopersplainsensis* type sequence (EU111770) (Fig. 10).

#### *Francisella* and *Mycoplasma*

*Francisella* was identified in four ticks, *Am. triguttatum* (three) and *Ix. australiensis* (one) from brush-tailed possums and quenda in Western Australia (Table 4, Supplementary file S2). *Mycoplasma* was present in three samples, from three individuals: two blood samples from a black rat and a swamp rat and one tissue sample from a black rat (Table 4, Supplementary file S2).

## Discussion

This body of work represents the first large-scale investigation into tick (and vector)-associated bacteria concurrently with their vertebrate hosts in urban and peri-urban locations, and identified novel tick-associated bacteria in Australian wildlife. Bacterial amplicon sequencing showed that there were limited taxa shared between blood, tick and tissue samples. The identification of bacterial taxa of interest was generally confined to certain hosts and/or sample types. To the best of the authors’ knowledge, this study represents the first report of *Neoehrlichia* and *Ehrlichia* from Australian wildlife (blood and/or tissue samples). The first identification of the rodent-associated *Borrelia* clade in Australia is also described. These findings show that geographical boundaries and host associations influence the prevalence of tick-associated bacteria. Importantly, these findings also provide support for the absence of northern hemisphere tick-borne pathogens, adding to the overwhelming body of evidence that any potential human tick-borne pathogens in Australia are probably endemic and genetically distinct from currently described pathogens in parts of North America and Europe.

Although a comparatively deep level of sequencing was achieved compared to similar studies [64, 65], rarefaction plots showed that blood samples did not reach the same asymptote as tick and tissue, indicating that deeper sequencing of these samples is needed to ensure complete coverage of bacterial diversity. Across all four alpha diversity measures, tissue samples showed the highest diversity and variance. In comparison, tick samples showed a less diverse bacterial composition. This is likely to be due to the dominance of endosymbiont bacteria in the tick microbiome, which has been demonstrated in previous studies of the same tick species as identified in the present study [17, 18], and in tick species globally [66, 67]. The overall lack of similarity in bacterial communities between sample types observed in the present work has been reported in previous studies on small mammals and their vectors (ticks and fleas), indicating the complexity of microbial assemblages inhabiting these biological niches [65, 68, 69]. The diagnostic value of 16S rRNA amplicon sequencing methods has not been thoroughly validated for tick-borne pathogens. One study, however, showed that ticks which tested positive for *Borrelia* using quantitative PCR methods also had a high abundance of *Borrelia* using amplicon bacterial metabarcoding [70]. Therefore, even though *Ix. holocyclus* and *Ix. tasmani* had abundant endosymbionts, and the presence of bacterial species in lower abundance cannot be ruled out completely, it is likely that our finding of the absence of some bacterial species here reflects the true situation.

The microbiome of *Ix. holocyclus* and *Ix. tasmani* was dominated by a limited number of highly abundant ASVs. Other tick species, such as *Ix. trichosuri* and *Ix. australiensis*, showed a more diverse bacterial composition. The interaction between bacterial endosymbionts and pathogens in ticks is not yet fully understood. The presence of endosymbiont bacteria is thought to inhibit the colonization by other potentially pathogenic bacterial species. For example, colonization by the rickettsial endosymbiont (* Rickettsia peacockii *) in *Dermacentor andersoni* tick ovaries is thought to render the ticks more resistant to co-infection by a second rickettsial species (* Rickettsia rickettsii *), which is the main cause of Rocky Mountain Spotted Fever [71]. Likewise, *Dermacentor variabilis* ticks infected with one strain of *Rickettsia* were shown to be less susceptible to secondary rickettsial infections [72]. However, this theory is not uniformly accepted, and there is some rejection of Burgdorfer’s [71] initial findings. Instead, it is hypothesized that differences in *R. peacockii* prevalence in *De. andersoni* are due to microclimatic factors [73]. Additionally, in recent years microbiome profiling has led to conflicting conclusions about the interaction between endosymbiont and pathogens [74]. Based on what is known from the northern hemisphere examples, tick species without a dominant endosymbiont may pose a greater risk of harbouring potentially pathogenic microbes [74].

Equivalent studies from the northern hemisphere using 16S rRNA metabarcoding methods and wildlife surveillance to identify tick-borne pathogens are limited. Instead, pathogen surveillance studies are generally targeted to well-known pathogens. For example, studies of *B. burgdorferi sensu lato* have shown infection is not detrimental to the health of natural rodent reservoirs [75], which have been shown to harbour a diverse range of *Borrelia* species [76]. In the present study, chuditch and long-nosed bandicoots had a high abundance of *Anaplasmataceae* in blood samples, while brush-tailed possums had the highest abundance of this taxon in tissue samples. In addition, *Anaplasmataceae* bacteria were mostly absent from larval ticks. This suggests that the *Anaplasmataceae* bacteria identified are probably maintained via horizontal transmission (i.e. between host and tick) and transstadial routes, which has been observed with related bacteria in companion animals [77, 78]. In contrast, the identification of *Midichloria* across all life stages of *Ix. holocyclus* shows evidence of vertical (transovarial) transmission. Both horizontal and vertical transmission have been identified previously in other tick species with *Midichloria* [79, 80]. The absence of *Midichloria* in vertebrate hosts (blood and skin) in the present study suggests the horizontal infection route is not a major contributor to the presence of this endosymbiont in *Ix. holocyclus* and is consistent with previous studies [81].

### Taxa of interest

#### Anaplasmataceae

The present study has provided the first identification of *Neoehrlichia* spp. from Australian wildlife. Two *Neoehrlichia* species, ‘*Ca*. N. arcana’ and ‘*Ca*. N. australis’, were recently characterized from *Ix. holocyclus* along the east coast of Australia at a prevalence of 11.3% (44/391) [24]. This finding is consistent with other species of *Neoehrlichia* globally, which have been described circulating in a range of mammals and associated ticks [82, 83]. In contrast to Gofton *et al*. [24], the results presented here show that ‘*Ca*. N. arcana’ is more prevalent than ‘*Ca*. N. australis’ in small mammals, which is consistent with a recent study of Australian wildlife ticks [18]. The main reservoir host for ‘*Ca*. N australis’ is likely to be medium to large macropods [e.g. eastern grey kangaroo (*Macropus giganteus*) or swamp wallaby (*Wallabia bicolor*)], which were not sampled in the present study.

The identification of three putative novel *Anaplasmataceae* species further demonstrates the high diversity of *Anaplasmataceae* from Australian ticks and wildlife. This finding adds to recent discoveries identifying new species from *Am. triguttatum* [23], *Bothriocroton concolor* [84], *Ix. holocyclus* [24], and from the platypus (*Ornithorhynchus anatinus*) and its associated ticks *Ix. ornithorhynchi* [85]. The novel *Anaplasmataceae* species were less readily identified in tick vectors compared to ‘*Ca*. N. arcana’ and ‘*Ca*. N. australis’; however, more targeted studies are needed to understand their prevalence and distribution.

The first identification of *Anaplasma platys* (formerly *Ehrlichia platys*) in Australia was made in 2001 [86], and has since been described from dogs throughout the country [87, 88]. To date it has not been identified in any native Australian wildlife, although *A. platys* has been reported from deer overseas [89]. To the best of our knowledge, this is the first report of *A. platys* from wild deer in Australia. The vector of *A. platys* overseas, *Rhipicephalus sanguineus* [90], is probably also the vector in Australia. The brown dog tick, syn. *Rh. sanguineus,* tropical lineage was recently named *Rhipicephalus linnaei* [91]. Importantly, *Ehrlichia canis *, which was first identified in dogs and brown dog ticks in northern Australia during 2020, was not identified in ticks, tissue or blood from wildlife in this study.

#### Midichloria

While ‘*Ca*. M. mitochondrii’ (CMm) is known to be highly prevalent and abundant in ticks identified in the present study, the present study did not employ a blocking primer as used in previous studies [17, 18]. The justification for this was a deliberate choice to include CMm in the ‘taxa of interest’ investigation, i.e. provide insight into the ecology of this microbe. The absence of CMm in both blood and tissue was surprising, especially given the high proportion and abundance of the bacteria in *Ix. holocyclus* in the present study. Studies in France have identified CMm from roe deer (*Capreolus capreolus*) blood samples using molecular and serology techniques [80]; while that study reported a higher sensitivity for serology, molecular methods were also successful at identifying infection. *In vitro* studies of *Ix. ricinus* and rabbits suggest that the endosymbiont replicates within the vertebrate host [92]. The bacterial microbiome of many tick species is generally dominated by the presence of one (or a few) highly abundant endosymbiont microbes [17, 93, 94]. This suggests that rare, less abundant bacteria are not easily identified using standard 16S rRNA bacterial metabarcoding methods. While the possibility cannot be ruled out, given the high abundance of CMm in tick samples, it is likely that the methods employed here would have identified it in wildlife hosts (either blood or tissue) if present. Therefore, given the absence of CMm in the hosts sampled in the present study, vertical transmission may be the primary source of *Midichloria* infection for *Ix. holocyclus*. Alternatively, the reservoir host for CMm may not have been sampled.

#### Coxiellaceae

All *Coxiellaceae* ASVs identified were most similar to members of the genus *Rickettsiella* and dominated the microbiome of *Ix. tasmani. Coxiella* was not detected in the present study. The absence of *Coxiella* from host blood and tissue samples probably reflects a low abundance of this bacteria in the small mammal hosts sampled. Serological methods are more commonly used to detect exposure to *C. burnetii* [95] but lack the ability to detect acute infections. A recent study using molecular techniques detected *C. burnetii* in kangaroo meat samples [96]. This suggests that animals sampled in the present study were negative for an active infection with *Coxiella *.

#### Rickettsiaceae

While several forms of rickettsiosis are amongst the few recognized human tick-borne diseases in Australia, remarkably little is known about the sylvatic life cycle of these intracellular bacteria. At least five agents of human rickettsial disease have been described in Australia [97], together with several additional species and genotypes with unknown pathogenicity [97–99]. Bandicoots and other small mammals have previously been described as reservoirs for Queensland tick typhus (* R. australis *), and *Ix. holocyclus* and *Ix. tasmani* are known vectors [100, 101]. Although the relationship between bandicoots and rickettsial infections is largely accepted, there has been little validation of this association since the early publications.

In the present study, the genus *Rickettsia* was predominately identified from ticks, with a low prevalence and abundance in four tissue samples and was absent from all blood samples. Areas sampled in Sydney’s northern beaches are endemic for Queensland tick typhus and spotted fever. Therefore, the absence of spotted fever or typhus group *Rickettsia* from mammal hosts in the present study was unexpected. Recent studies utilizing qPCR have reported *Rickettsia* in 15.4% (23/149) *Ix. holocyclus* from NSW [102] and 6.4% (13/203) ticks collected from wildlife in north-east Queensland [103]. Additional reports also recognize the presence of spotted fever group *Rickettsia* in Australian ticks [100, 104, 105].

The lack of *Rickettsia* identified in ticks may be due to the highly abundant endosymbionts *Midichloria* or *Coxiellaceae* in *Ix. holocyclus* and *Ix. tasmani* respectively. While there are limited comparable studies that have used bacterial metabarcoding on wildlife blood samples, previous research has shown that molecular methods successfully identify *Rickettsia* in reservoir hosts [106, 107, 108]. Flinders Island Spotted Fever, caused by *R. honei *, is associated with reptiles and the southern reptile tick *Bo. hydrosauri* [109]. A closely related strain, named *R. honei marmionii*, has been identified from various regions in Australia and is associated with *Haemaphysalis novaeguineae* [110, 111]. Interestingly no *Haemaphysalis* ticks were identified from hosts in the present study. Further investigation into the sylvatic cycle of *Rickettsia* in Australia would benefit from the inclusion of samples from reptile hosts and vertebrate hosts of *Haemaphysalis* ticks.

#### Borrelia

The novel *Borrelia* identified here came exclusively from black rats and was found at two sites in Sydney’s Northern Beaches area. All samples that tested positive were tissue, and no *Borrelia* sequences were identified from corresponding blood or tick samples.

The *Borrelia* species identified from black rats formed a clade with other described *Borrelia* species identified from rodents. *Borrelia* sp. R57 was identified from bank voles (*Myodes glareolus*; formerly *Clethrionomys glareolus*) and wood mice (*Apodemus sylvaticus*) in Spain at a prevalence rate of 8.5–12.0% using PCR methods [112]. That study identified serological cross-reactivity of this species with *B. burgdorferi sensu lato* using western blot testing. A follow-up study (in Spain) further identified the genotype from 24.5% (62/253) of rodent tissue samples [113]. Identification of similar sequences was subsequently made from black rats (*R. rattus*) in California, USA, with a prevalence of 43.5% (10/23) in tissue (skin) biopsy samples [114]. The absence of this *Borrelia* species from our tick samples is interesting and mirrors the previous studies in Europe and the USA [112, 114]. Despite multiple attempts during this study, amplification at the *flaB* locus was unsuccessful, consistent with previous research of members within the clade [112].

The extensive geographical range of this clade, yet tight host associations identified to date (i.e. confined to the order Rodentia), raises questions regarding its origin. Particularly interesting is the paucity of reports globally, despite small mammals being the most widely studied reservoir host of tick-borne diseases [115]. Our phylogenetic analysis shows strong support for these *Borrelia* sequences forming a distinct clade and is consistent with other studies [116]. Its basal phylogenetic position may provide important insights into the evolutionary history of the genus *Borrelia *. The lack of its identification in tick samples means that the vector for this *Borrelia* sp. remains unknown, and the transmissibility and pathogenicity of this microbe requires further research. Importantly, no identifications were made of the recently characterized *Borrelia* species from Australia, ‘*Ca*. B. tachyglossi’ [21] or reptile *Borrelia* sp. [22], nor any sequences from the *B. burgdorferi sensu lato* group.

Currently there is no evidence that the novel rodent-associated *Borrelia* genotype identified in the present study is transmissible to humans or capable of causing disease. Phylogenetic reconstruction indicates that it is distinct from the *B. burgdorferi sensu lato* and relapsing fever groups [116]. The absence of detection from tick samples and its identification only in rodents more probably implies another vector that presumably has a tight host relationship with *Rattus* species.

#### Bartonella

*Bartonella* spp. were identified in a relatively high proportion of wildlife blood samples, and most prevalent in blood samples from black rats (31.0%). *Bartonella coopersplainsensis* and *Ba. queenslandensis* were described from native Australian rodents (*Melomys* spp. and *Rattus* spp.) in Queensland [117]. Since then, a number of novel *Bartonella* strains have been identified from Australia, including novel species described from marsupials and their ectoparasites (ticks and fleas) [118–120]. While generally considered to be transmitted by fleas, reports of *Bartonella* species isolated from ticks are increasing, as studies take a broader approach to surveillance of vector-associated microbes [65, 121, 122]. Identification of *Bartonella* in fed ticks does not mean they are a viable vector, but it does highlight that future studies should include ticks when exploring candidate vectors. The high prevalence of identification of *Bartonella* in rodents has been reported in other metabarcoding studies from blood samples [69].

#### *Francisella* and *Mycoplasma*

*Francisella* was identified exclusively from ticks in Western Australia, *Am. triguttatum* and *Ix. australiensis*. Previous bacterial profiling of *Am. triguttatum* has shown a high proportion of *Francisella* species [18, 123]. Given its high abundance in tick samples, the absence of *Francisella* in a corresponding host may indicate a lack of transmission. Tularemia (caused by *Francisella tularensis *) can cause disease in humans and animals. While the infection is more commonly acquired via handling infected animal material or aerosol inhalation, *F. tularensis* has been reported in ticks [124]. *Francisella* has been reported in several tick species [125]. While the species identified in the present study is not closely related to *F. tularensis *, considering the significance of this genus [124], further characterization should be of high importance.

The present study did not identify any haemoprotic mycoplasma species. The *Mycoplasma* sequences identified were most closely related to *Mycoplasma arthritidis *, which is recognized as a common pathogen in rats [126]. It was identified in two black rats and a single identification in the native swamp rat.

### Limitations and recommendations

While the cost of next-generation or second-generation sequencing platforms continues to decline, analysis of large numbers of samples is still constrained by resources and funds. Amplicon high-throughput sequencing remains a cost-effective approach for characterizing bacterial microbiomes. With the advent of long-read sequencing technologies (e.g. PacBio), more studies are moving towards obtaining full-length 16S rRNA sequences which allow for more robust phylogenetic reconstruction and in many cases identification of bacteria to strain/genotype level [127, 128]. In addition to describing the bacteria present, the idea of the functional tick microbiome has been suggested [129]. While adaptation of these methods can seem promising, their use outside of human microbiome studies is questionable due to the reliability of databases [74, 130]. An alternative approach such as the ‘common core’ or ecological ‘core microbiome’ may be more suitable in the context of tick microbiome research [131]. Studies incorporating transcriptomics will probably also prove useful for understanding the functional components of the microbiome.

While the present study reported on the relative abundance of microbes between ticks sampled, it is well known that differences in the 16S copy number limit inferences [132, 133]. In the context of the present study, relative abundance of taxa was compared among samples, as it was considered a suitable measure given samples were processed in the same manner. Calculations of absolute abundance using 16S rRNA metabarcoding are challenging. While copy number is one factor that can be modelled, it relies on information that in many cases is not accurately known. Using mock communities can help assess bias without baseline data on copy number, but these analysis methods are still limited.

The sensitivity of molecular diagnostic testing for tick-borne pathogens has been shown to vary greatly. Factors that can influence molecular detection of pathogens include level of parasitaemia/bacteraemia, sample type, time of sample collection from initial infection, and pre-analytical preparation techniques; additionally, this is compounded by differences between taxa due to host–pathogen interactions [134–136]. A strong advantage of using generic metabarcoding assays is their unbiased approach; in the context of the present study, the most important factor was to achieve unbiased characterization of bacterial communities from Australian wildlife. However, it is acknowledged that the use of bacterial metabarcoding for the identification of low-abundance and rare organisms is limited. The lack of recognized endemic bacterial pathogens such as *Coxiella burnetii* and *Rickettsia* spp. was surprising. Future studies should use serological and culture methods in addition to these highly specific and sensitive molecular tools (e.g. qPCR).

Another limitation of the present study was the bias in animals sampled. Small mammals were chosen as it was hypothesized they would have the highest prevalence of potential tick-borne pathogens based on what is known from the northern hemisphere [137–139]. Further sampling with expanded host and spatial aspects are vital to understand the full epidemiology of tick- (and vector) associated microbes. Likewise, alternative trapping methods (e.g. differing trap type, bait) may expand the suite of hosts captured, importantly those with territorial overlap (e.g. semi-arboreal marsupials).

In the present study, most taxa of interest were confined to specific sample types. For example, *Borrelia* was identified from tissue samples of rats but absent from any blood or tick samples. This finding has also been reported in white-footed mice (*Peromyscus leucopus*) [140]. While in many cases disease surveillance is targeted to known pathogens where sampling protocols and diagnostics are well defined, in scoping studies such as the present one, a range of sample types are essential. This is evident in the different ways pathogens act within their host. As the first line of defence, the innate immune system has a key role in protecting against cutaneous and systemic infection [141]. Alternatively pathogens can quickly evade host innate immune responses by rapidly disseminating into the bloodstream after invasion [142, 143]. In the context of Australia, however, many wildlife species are endangered and therefore their capture and sampling in the wild is subject to strict ethical conditions and government regulations. As such, collection of multiple sample types is restricted to those requiring only a minimally invasive method and is also dependent on the availability of trained personnel and resources. In many instances it is standard procedure to collect a tissue biopsy for animal genetic (population) purposes, and this presents a simple alternative to other procedures which may require field anaesthesia. The results here highlight the value of this sample type for use in pathogen surveillance.

## Conclusions

Increased land-use and climate change will continue to affect the spread of vectors and associated diseases globally. There is an urgent need for expanded disease surveillance, particularly at the urban interface with wildlife. Through collaborative approaches, discoveries of novel microbes and important epidemiological links will be critical to ensure adequate and timely responses to human and animal diseases. Investigations into taxa of interest in the present study showed *Anaplasmataceae* bacteria had a relatively high prevalence in the vertebrate hosts examined here and a wide geographical distribution. This group of bacteria is therefore a viable candidate for continued research into Australian zoonotic tick-borne diseases.

## Supplementary Data

Supplementary material 1
