# Supplementary material for: The bacterial biome of ticks and their wildlife hosts at the urban–wildland interface
Source: Microb Genom. 2021 Dec 16;7(12):000730. doi: 10.1099/mgen.0.000730 (PMC8767321; doi:10.1099/mgen.0.000730)
Supplement: Supplementary material 1 [file mgen-7-0730-s001.pdf]

SUPPLEMENTARY MATERIAL

The bacterial biome of ticks and their wildlife hosts at the urban-wildland interface

Microbial Genomics, 2021

Authors: Siobhon L. Egan[#], Casey L. Taylor, Peter B. Banks, Amy S. Northover, Liisa A. Ahlstrom, Una M. Ryan, Peter J. Irwin, Charlotte L. Oskam[#]

# Correspondence: siobhon.egan@murdoch.edu.au; c.oskam@murdoch.edu.au

---

Supplementary file 1 – this file contains additional tables, figures and statistical analysis.

- Table S1
- Figures S1-S10
- Statistical analysis results

Supplementary files 2–5 – these files are electronic material that are available for download on FigShare, repository doi: 10.6084/m9.figshare.14363627.

Supplementary file 1

Table S1: Summary of sequences obtained from bacterial next-generation sequencing

Bioinformatics step	Control (totals)	Sample (totals)	Blood	Tick	Tissue
Sum input	5534288	96969355	35160160	24844688	36964507
Mean input	85143	152228	185053	95556	197671
Sum filtered	3922421	70048790	24929182	19809370	25310238
Mean filtered	60345	109967	131206	76190	135349
Filtered %	70.90%	72.20%	70.90%	79.70%	68.50%
Sum denoised	3906305	69477650	24758558	19753200	24965892
Mean denoised	60097	109070	130308	75974	133507
Sum merged	3815914	67183779	24028959	19127172	24027648
Mean merged	58706	105469	126468	73566	128490
Merged %	69.00%	69.30%	68.30%	77.00%	65.00%
Sum non-chimeric	3719634	64186307	23061496	18368918	22755893
Mean non-chimeric	57225	100763	121376	70650	121689
Input non-chimeric %	67.20%	66.20%	65.60%	73.90%	61.60%

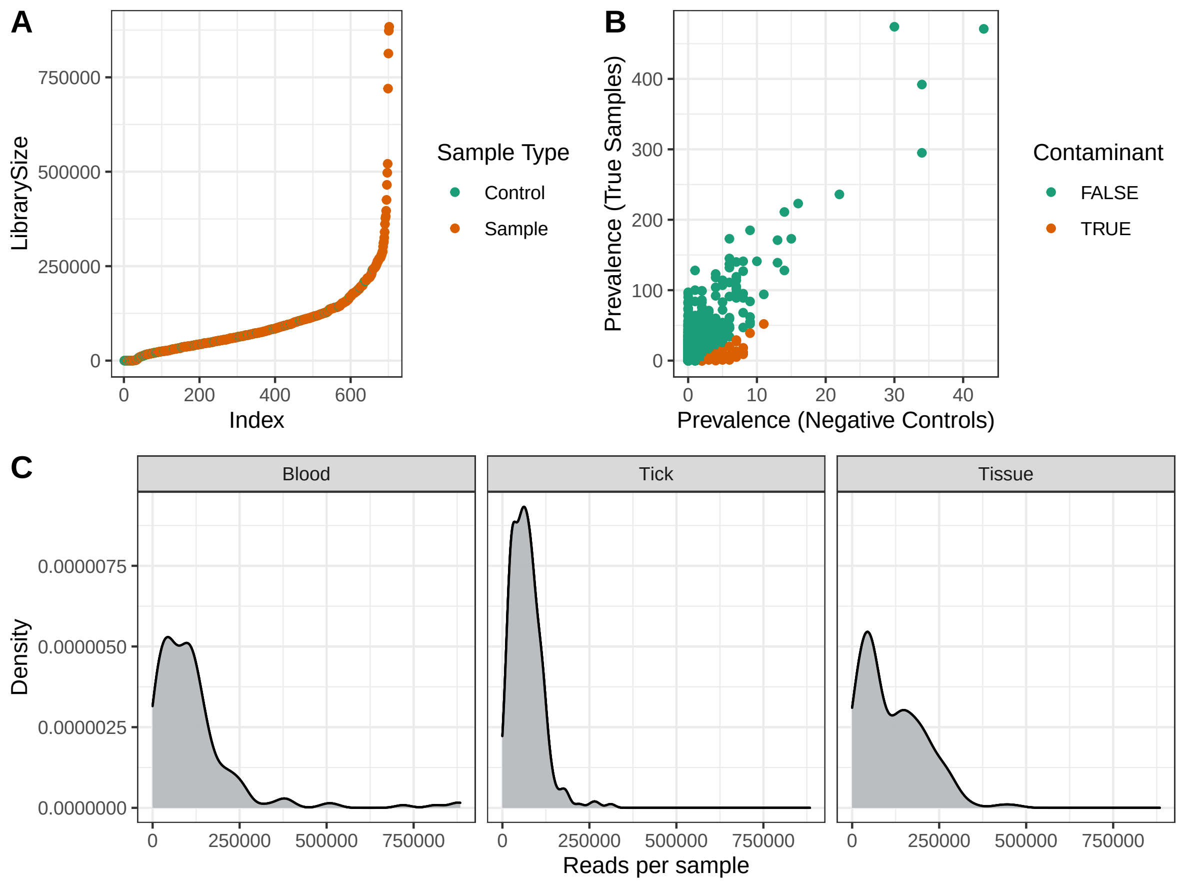

Figure S1: Summary of sequences obtained form bacterial 16S rRNA next-generation sequencing (Illumina MiSeq). (A) Library size (i.e. number of sequences) obtained from samples. (B) Prevalence of contaminant ASVs as identified by ‘decontam‘ analysis in controls and samples. (C) Distribution of the number of reads in sample categories blood, tick and tissue.

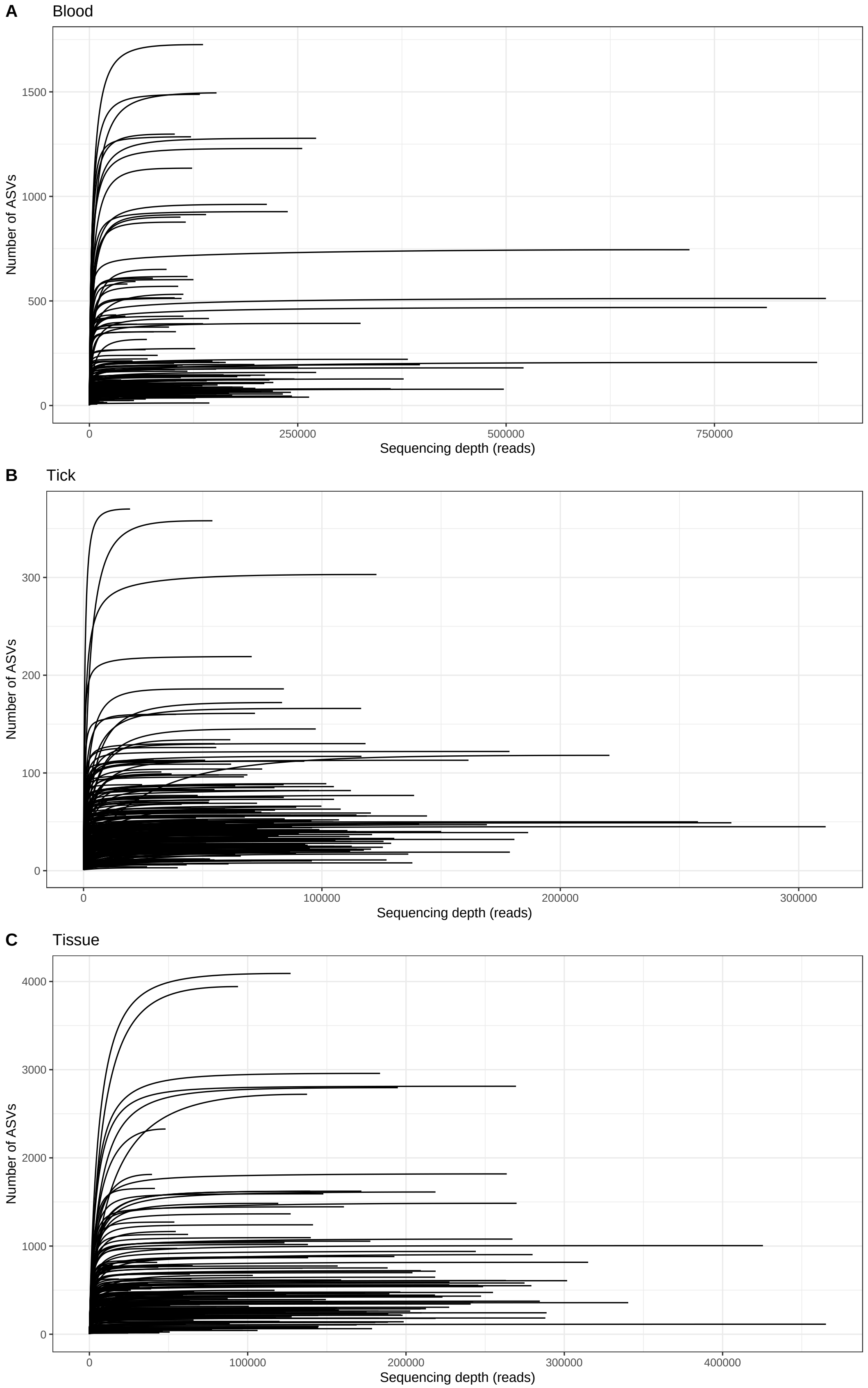

Figure S2: Rarefaction curves (number of ASVs) of wildlife blood, tick and tissue samples from bacterial 16S rRNA profiling (step size = 100). Rarefaction curves were used to determine how adequate sequencing depth was in detecting the complete theoretical suite of bacterial organisms present; of note, rarefaction plots excluded OTUs considered environmental contaminants (described methods).

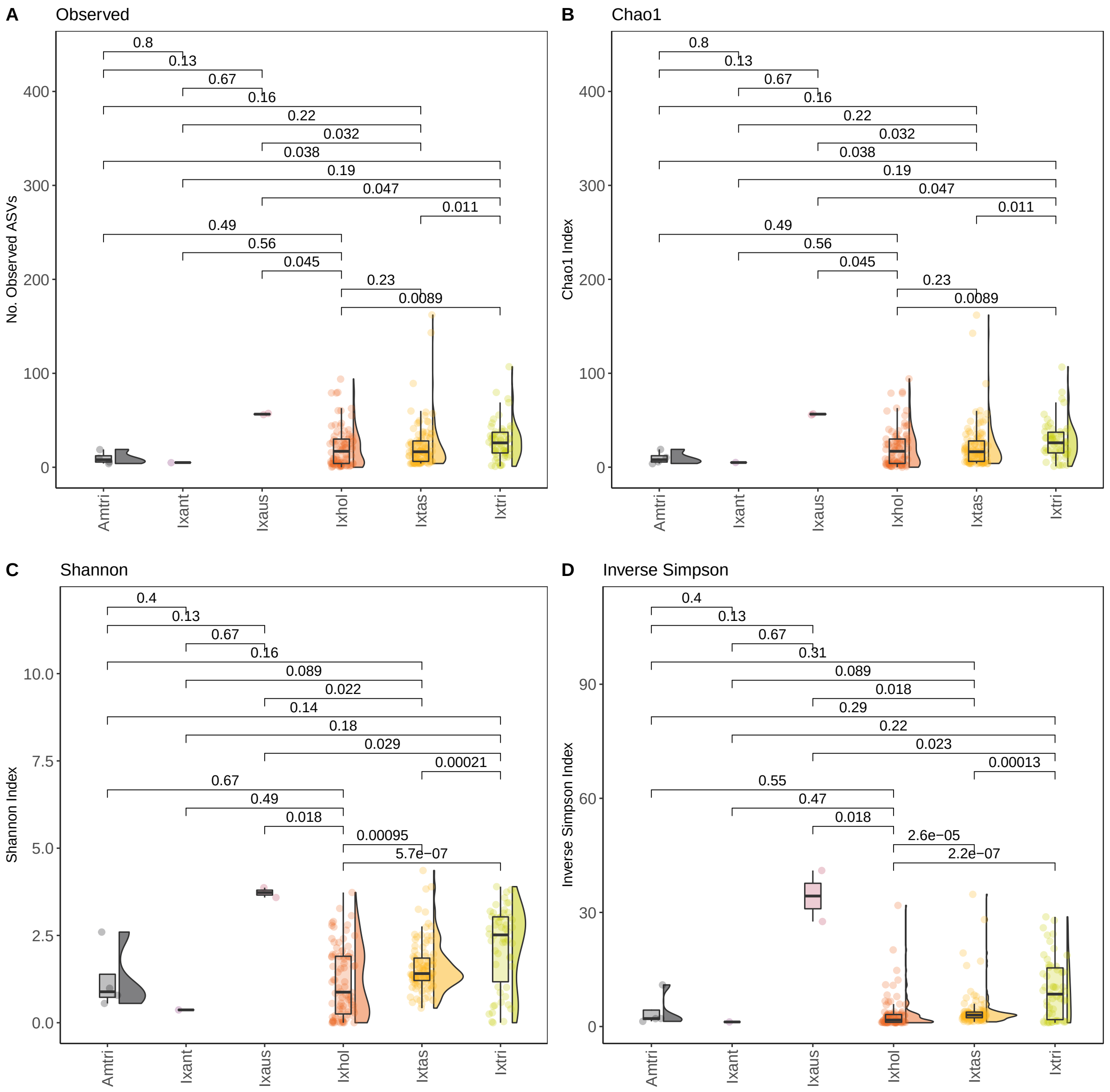

Figure S3: Boxplot of Alpha-diversity indices for tick samples. Diversity indexes (A) Observed number of ASVs, (B) Chao1 index, (C) Shannon index and (D) inverse Simpson index. Boxplots and violin plots represent the distribution of diversity among tick species. Statistical analysis between sample types calculated using Wilcoxon pairwise (non-parametric) test. Pools of different tick species excluded from analysis.

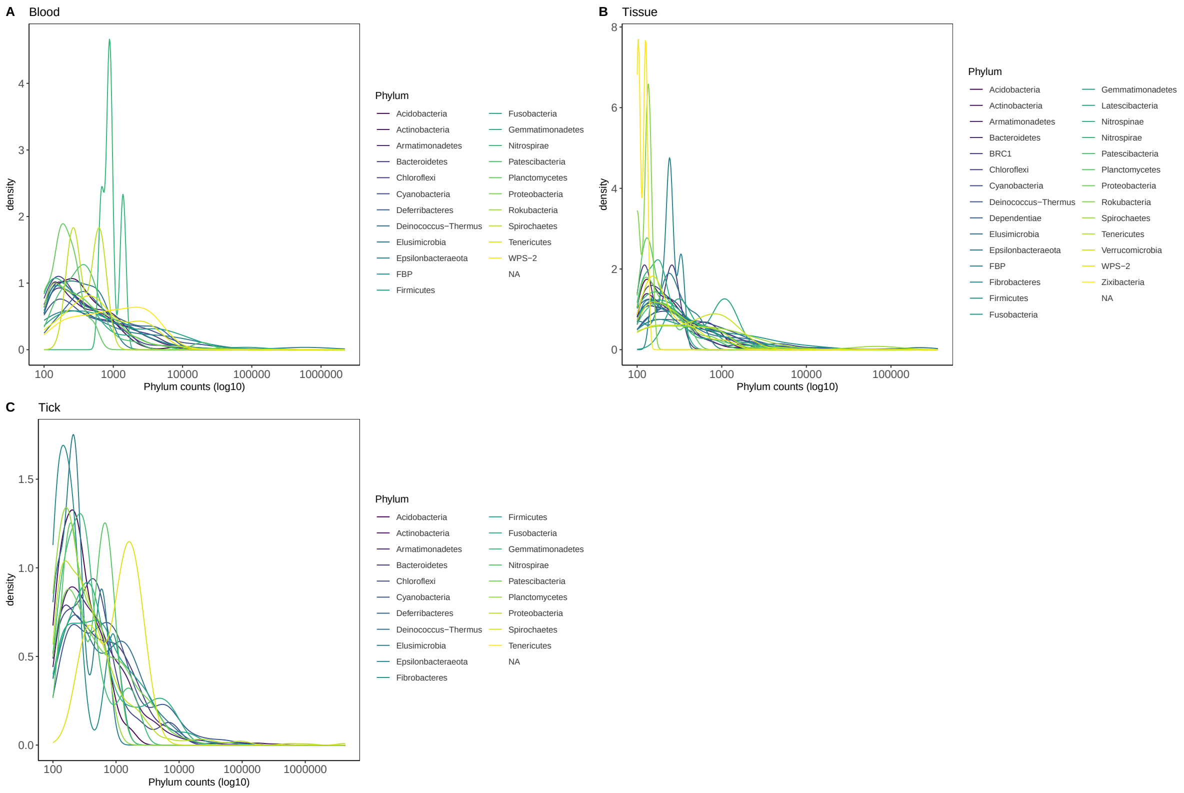

Figure S4: Phylum level distribution plots of bacterial composition in wildlife samples.

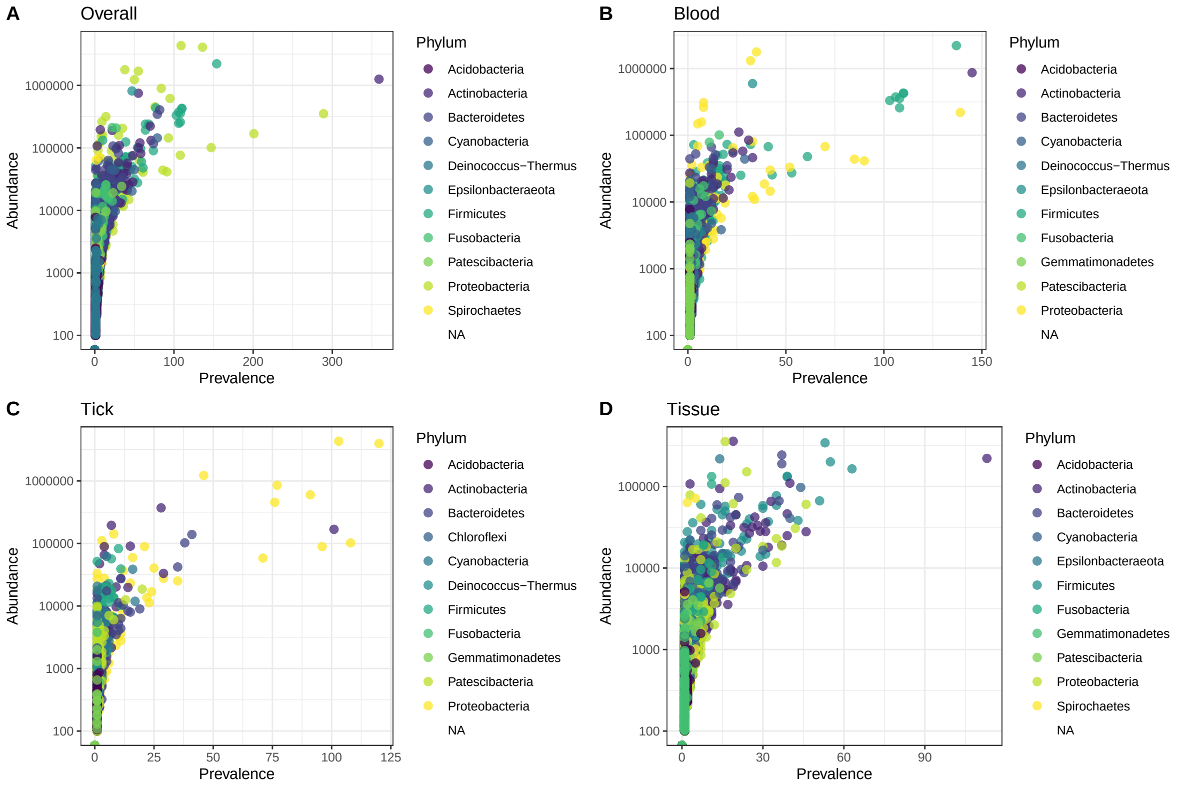

Figure S5: Abundance (no. of sequences) and prevalence in samples of bacterial phyla identified from wildlife samples (A) overall, (B) blood, (C) tick and (D) tissue.

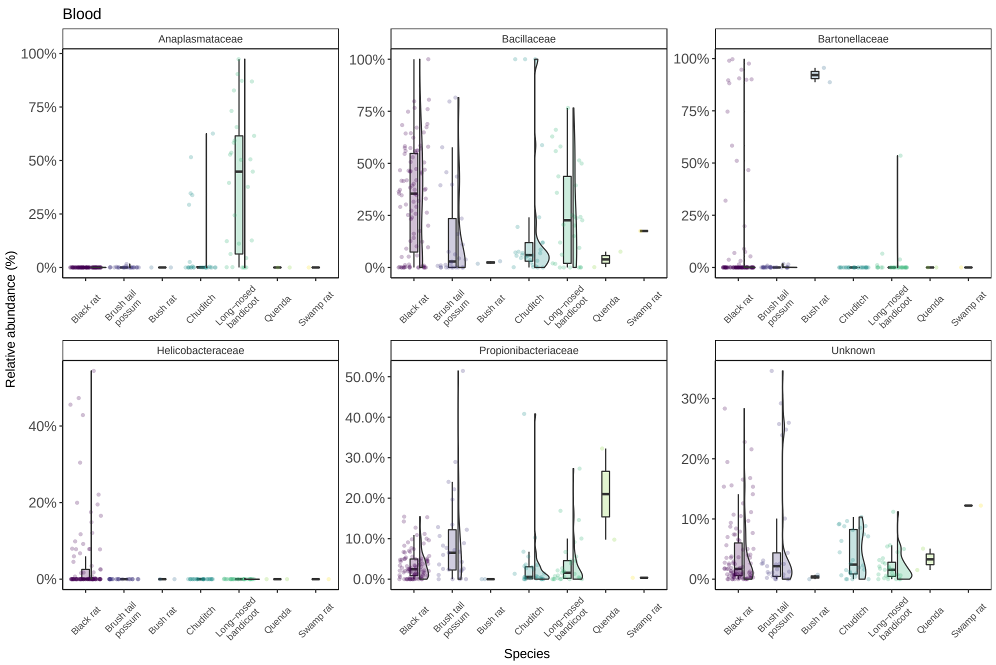

Figure S6: Bacterial family taxa (top 6 most abundant) identified in wildlife blood samples.

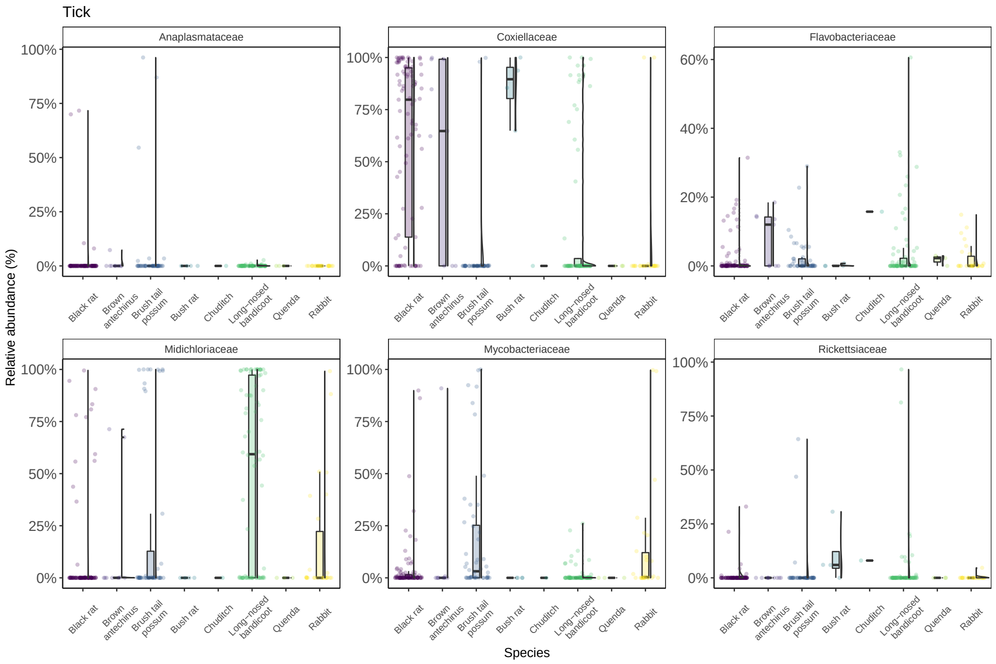

Figure S7: Bacterial family taxa (top 6 most abundant) identified in wildlife tick samples.

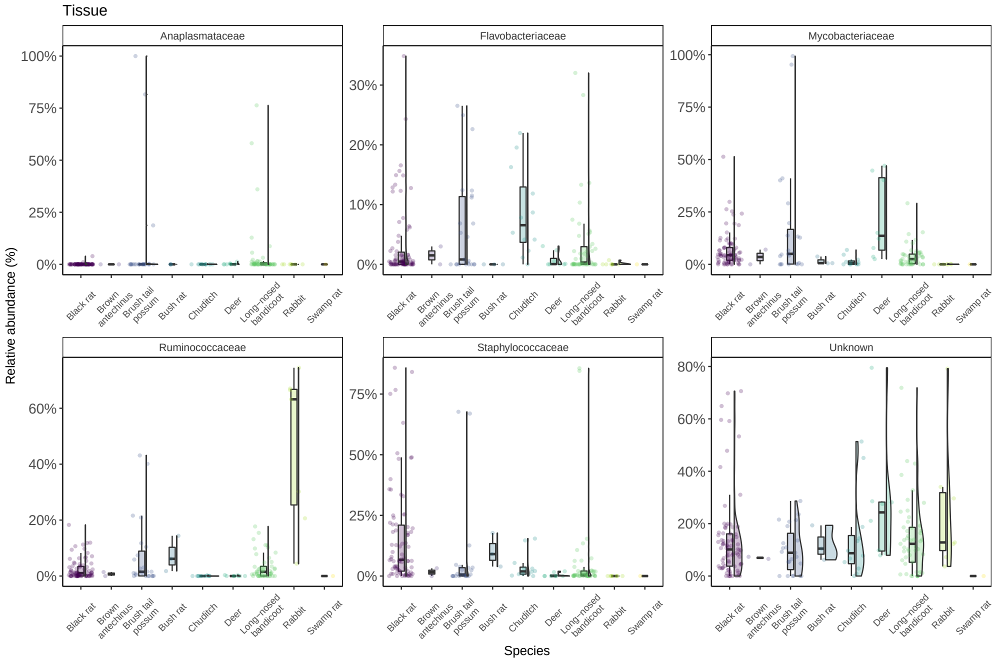

Figure S8: Bacterial family taxa (top 6 most abundant) identified in wildlife tissue samples.

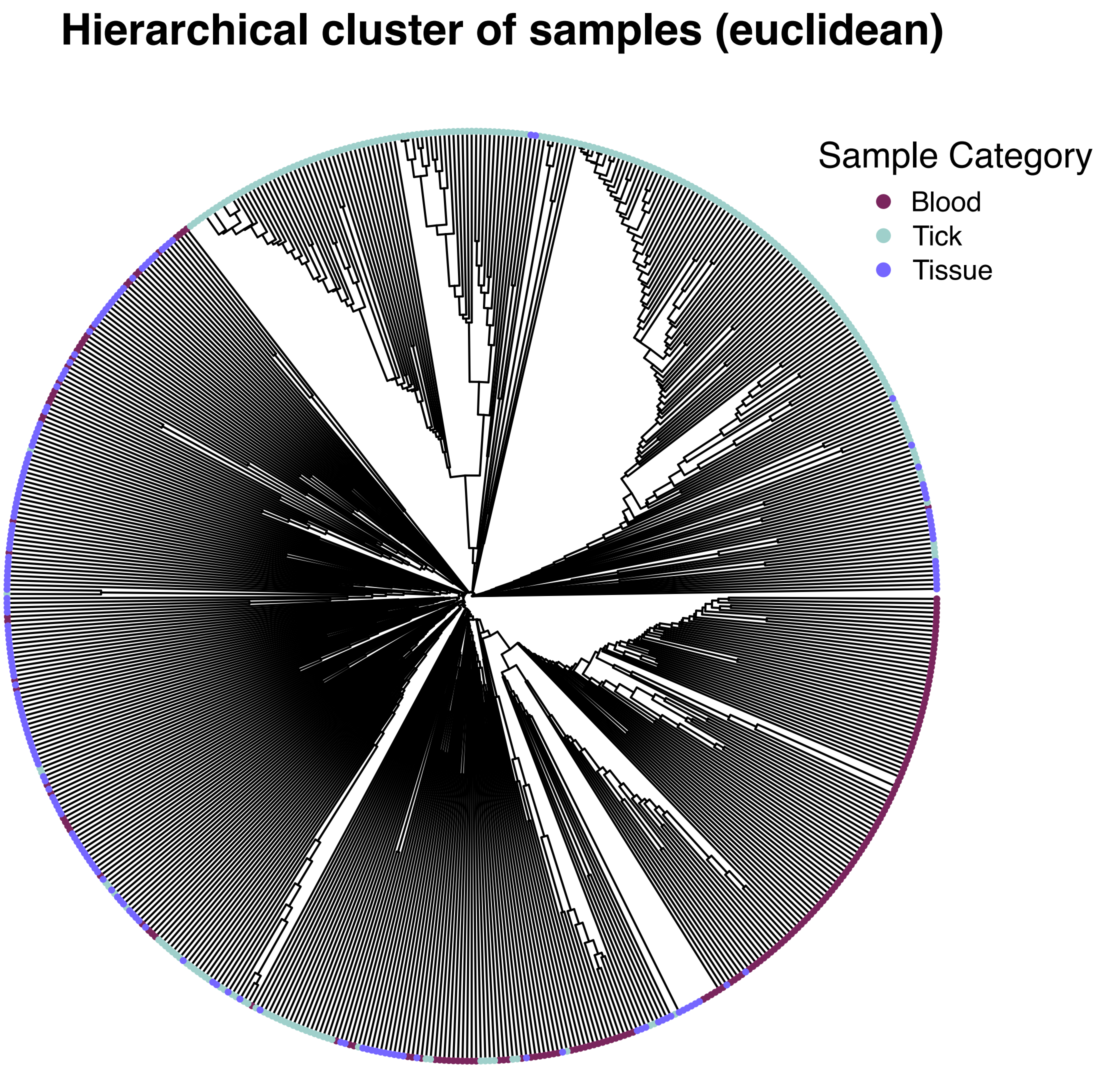

Figure S9: Hierarchical cluster analysis of bacterial communities from wildlife samples. Data points coloured by sample type; blood, tick and tissue. Cluster analysis was performed using euclidean distance measure (average), data was transformed using Hellinger transformation.

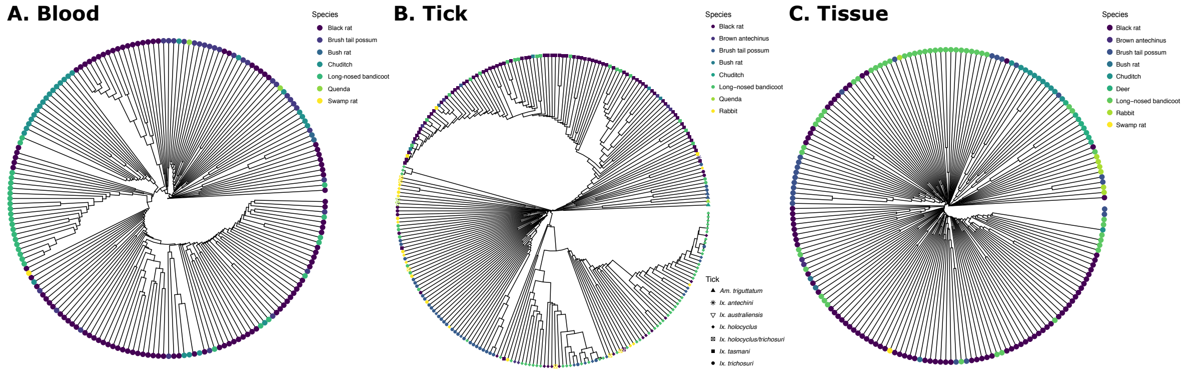

Figure S10: Hierarchical cluster analysis of bacterial communities from wildlife samples, separated by sample types. (A) Blood, (B) Tick, (C) Tissue. Data points coloured by host species (and tick species (B)). Cluster analysis was performed using euclidean distance measure (average), data was transformed using Hellinger transformation.

Statistical analysis

Beta diversity statistical analysis of bacterial microbiome composition using analysis of variance (ANOVA) and permutational multivariate analysis of variance (PERMANOVA) methods. Statistical analysis calculated using the vegan R package. Data cleaning and transformation: removed samples with < 1000 sequences, transformed data using Hellinger transformation method. Distance calculated using Bray-Curtis distance measure.

Sample Type

Statistical analysis comparing the bacterial microbiome of blood, tick and tissue samples collected form wildlife.

A. Analysis of Variance (ANOVA)

```
Analysis of Variance Table

Response: Distances
           Df Sum Sq  Mean Sq F value    Pr(>F)
Groups      2 0.4466 0.223289  36.209 1.364e-15 ***
Residuals 614 3.7863 0.006167
---
Signif. codes:  0 ‘***’ 0.001 ‘**’ 0.01 ‘*’ 0.05 ‘.’ 0.1 ‘ ’ 1
```

B. Permutational multivariate analysis of variance (PERMANOVA)

```
Permutation test for homogeneity of multivariate dispersions
Permutation: free
Number of permutations: 999

Response: Distances
           Df Sum Sq  Mean Sq      F N.Perm Pr(>F)
Groups      2 0.4466 0.223289 36.209    999  0.001 ***
Residuals 614 3.7863 0.006167
---
Signif. codes:  0 ‘***’ 0.001 ‘**’ 0.01 ‘*’ 0.05 ‘.’ 0.1 ‘ ’ 1

Pairwise comparisons:
(Observed p-value below diagonal, permuted p-value above diagonal)
            Blood       Tick Tissue
Blood             4.1600e-01  0.001
Tick   4.0839e-01             0.001
Tissue 8.8347e-17 3.5464e-15
```

Tick species

Statistical analysis comparing bacterial microbiome of tick collected from wildlife (all tick species).

A. Analysis of Variance (ANOVA)

```
Analysis of Variance Table

Response: Distances
           Df Sum Sq Mean Sq F value    Pr(>F)
Groups      6 2.5929 0.43215   14.01 9.594e-14 ***
Residuals 251 7.7423 0.03085
```

B. Permutational multivariate analysis of variance (PERMANOVA)

```
Permutation test for homogeneity of multivariate dispersions
Permutation: free
Number of permutations: 999

Response: Distances
           Df Sum Sq Mean Sq     F N.Perm Pr(>F)
Groups      6 2.5929 0.43215 14.01    999  0.001 ***
Residuals 251 7.7423 0.03085
---
Signif. codes:  0 ‘***’ 0.001 ‘**’ 0.01 ‘*’ 0.05 ‘.’ 0.1 ‘ ’ 1

Pairwise comparisons:
(Observed p-value below diagonal, permuted p-value above diagonal)
                 Amtri Ixant      Ixaus      Ixhol Ixhol;Ixtri      Ixtas Ixtri
Amtri                        9.4400e-01 3.6500e-01  2.5700e-01 7.8500e-01 0.001
Ixant
Ixaus       9.3797e-01                  5.9300e-01  3.8000e-02 7.3300e-01 0.001
Ixhol       3.4424e-01       5.7323e-01             5.3700e-01 1.0000e-03 0.001
Ixhol;Ixtri 2.4249e-01       4.5601e-02 5.3705e-01             3.8000e-02 0.001
Ixtas       7.8073e-01       7.4761e-01 3.5985e-05  3.8405e-02            0.001
Ixtri       1.0858e-06       4.6854e-09 2.0461e-06  1.2151e-05 5.1524e-15
```

Statistical analysis comparing bacterial microbiome of cohabiting tick species collected from wildlife in Sydney Northern Beaches area, New South Wales (*Ix. holocyclus*, *Ix. tasmani*, and *Ix. trichosuri*).

A. Analysis of Variance (ANOVA)

```
Analysis of Variance Table

Response: Distances
           Df Sum Sq Mean Sq F value    Pr(>F)
Groups      3 2.3202 0.77341  25.465 2.177e-14 ***
Residuals 247 7.5019 0.03037
---
Signif. codes:  0 ‘***’ 0.001 ‘**’ 0.01 ‘*’ 0.05 ‘.’ 0.1 ‘ ’ 1
```

B. Permutational multivariate analysis of variance (PERMANOVA)

```
Permutation test for homogeneity of multivariate dispersions
Permutation: free
Number of permutations: 999

Response: Distances
           Df Sum Sq Mean Sq      F N.Perm Pr(>F)
Groups      3 2.3202 0.77341 25.465    999  0.001 ***
Residuals 247 7.5019 0.03037
---
Signif. codes:  0 ‘***’ 0.001 ‘**’ 0.01 ‘*’ 0.05 ‘.’ 0.1 ‘ ’ 1

Pairwise comparisons:
(Observed p-value below diagonal, permuted p-value above diagonal)
                 Ixhol Ixhol;Ixtri      Ixtas Ixtri
Ixhol                   5.3000e-01 1.0000e-03 0.001
Ixhol;Ixtri 5.3687e-01             3.9000e-02 0.001
Ixtas       3.5862e-05  3.8383e-02            0.001
Ixtri       2.0485e-06  1.2237e-05 5.1829e-15
```

The following electronic supplementary files are available for download on FigShare, repository doi: 10.6084/m9.figshare.14363627.

Supplementary file 2

Supplementary file 2. Detail for taxa of interest with sequence information and sample metadata, including results from NCBI BLAST analysis. Taxa of interest sample data and BLAST results

Supplementary file 3

Supplementary file 3. Genetic similarity of Anaplasmataceae sequences based on a 1,244 bp alignment of the 16S rRNA locus. Sequences generated from the present study in bold. Bottom half of matrix showing sequence similarity and top half containing genetic distance data. Anaplasmataceae genetic diversity table

Supplementary file 4

Supplementary file 4. Genetic similarity of *Borrelia* sequences based on a 431 bp alignment of the 16S rRNA locus. Sequences generated from the present study in bold. Bottom half of matrix showing sequence similarity and top half containing genetic distance data. *Borrelia* genetic diversity table

Supplementary file 5

Supplementary file 5. Genetic similarity of *Bartonella* sequences based on a 522 bp alignment of the 16S rRNA-ITS locus. Sequences generated from the present study in bold. Bottom half of matrix showing sequence similarity and top half containing genetic distance data. *Bartonella* genetic diversity table
